# A high-throughput screen of inactive X chromosome reactivation identifies the enhancement of DNA demethylation by 5-aza-2′-dC upon inhibition of ribonucleotide reductase

**DOI:** 10.1186/s13072-015-0034-4

**Published:** 2015-10-13

**Authors:** Alissa Minkovsky, Anna Sahakyan, Giancarlo Bonora, Robert Damoiseaux, Elizabeth Dimitrova, Liudmilla Rubbi, Matteo Pellegrini, Caius G. Radu, Kathrin Plath

**Affiliations:** Department of Biological Chemistry, David Geffen School of Medicine, Eli and Edythe Broad Center for Regenerative Medicine, Jonsson Comprehensive Cancer Center, Molecular Biology Institute, University of California, 615 Charles E. Young Drive South, BSRB 390D, Los Angeles, 90095 USA; Department of Molecular and Medicinal Chemistry and California NanoSystems Institute, University of California, Los Angeles, 90095 USA; Ahmanson Translational Imaging Division, Department of Molecular and Medical Pharmacology, University of California, Los Angeles, 90095 USA; Department of Molecular, Cell, and Developmental Biology, University of California, Los Angeles, 90095 USA

**Keywords:** X chromosome inactivation, DNA methylation, 5-aza-2′-dC, Ribonucleotide reductase, Hydroxyurea

## Abstract

**Background:**

DNA methylation is important for the maintenance of the silent state of genes on the inactive X chromosome (Xi). Here, we screened for siRNAs and chemicals that reactivate an Xi-linked reporter in the presence of 5-aza-2′-deoxycytidine (5-aza-2′-dC), an inhibitor of DNA methyltransferase 1, at a concentration that, on its own, is not sufficient for Xi-reactivation.

**Results:**

We found that inhibition of ribonucleotide reductase (RNR) induced expression of the reporter. RNR inhibition potentiated the effect of 5-aza-2′-dC by enhancing its DNA incorporation, thereby decreasing DNA methylation levels genome-wide. Since both 5-aza-2′-dC and RNR-inhibitors are used in the treatment of hematological malignancies, we treated myeloid leukemia cell lines with 5-aza-2′-dC and the RNR-inhibitor hydroxyurea, and observed synergistic inhibition of cell growth and a decrease in genome-wide DNA methylation.

**Conclusions:**

Taken together, our study identifies a drug combination that enhances DNA demethylation by altering nucleotide metabolism. This demonstrates that Xi-reactivation assays can be used to optimize the epigenetic activity of drug combinations.

**Electronic supplementary material:**

The online version of this article (doi:10.1186/s13072-015-0034-4) contains supplementary material, which is available to authorized users.

## Background

X chromosome inactivation (XCI) is a program of transcriptional gene silencing that occurs on one of two X chromosomes in female mammalian cells to equalize gene dosage of X-linked genes to male cells. The inactive X chromosome (Xi) is a striking example of developmentally regulated heterochromatin formation in mammals. XCI has served as paradigm for understanding factors with generalized roles in gene silencing genome-wide such as DNA methylation and Polycomb protein-mediated histone methylation [1–3]. The Xi is established early in female embryonic development through a series of stepwise molecular changes that cooperate to ensure stable chromosome-wide gene silencing. Once established, the Xi is inherited through all somatic cell divisions and adult life [1–3]. XCI is initiated by the upregulation of the long noncoding RNA *Xist* from the maternal or paternal X chromosome early in embryonic development [1–3]. *Xist* coats the X chromosome from which it is expressed and initiates a cascade of events including exclusion of RNA polymerase II, changes in histone marks, and recruitment of structural chromosome proteins [1–3]. Accumulation of the histone variant macroH2A1 and gain of CpG island methylation characterize the transition to the maintenance phase of XCI, which is marked by resistance to X chromosome reactivation (XCR) upon deletion of *Xist* [4–9]. Thus, *Xist* is absolutely required for the initiation of XCI, but later is largely dispensable for the maintenance of the Xi, due to the presence of various other repressive chromatin marks [8, 9]. Notably, complete XCR is induced in vivo during pre-implantation and germ line development and in vitro by reversing cellular identity to the pluripotent state [10–13].

Despite the observation that many repressive chromatin factors are implicated in Xi establishment and maintenance, interference with DNA methylation has thus far shown the largest effect on eliciting loss of gene silencing on the Xi [5, 9, 14]. It is therefore thought that DNA methylation may uniquely ‘lock-in’ the silenced state and execute a greater influence on the robust nature of Xi maintenance than other repressive regulatory mechanisms [9]. DNA methylation concentrates on CpG islands in the course of XCI with redistribution away from intragenic and intronic CpGs relative to the active X chromosome [5, 7, 15, 16, 17]. CpG island methylation on the Xi is established by the de novo methyltransferase DNMT3B and is subsequently propagated by the maintenance methyltransferase DNMT1 [5, 9, 15]. Interference with DNA methylation by deletion of *Dnmt1* or treatment with 5-aza-2′-deoxycytidine (5-aza-2′-dC, also called decitabine) has been shown to induce the reactivation of an Xi-linked reporter gene and endogenous X-linked genes in a proportion of female somatic cells [9]. 5-aza-2′-dC is a deoxycytidine analog that upon phosphorylation incorporates into DNA and irreversibly inhibits DNMT1 [18]. Subsequent rounds of DNA replication therefore lead to passive DNA demethylation due to the absence of DNMT1 activity [19]. Together these findings indicate that Xi reporter systems permit the functional analysis of gene silencing, and that in addition to DNA methylation various other mechanisms contribute to Xi silencing. Therefore, XCI is an attractive model system to probe therapeutic approaches to the reactivation of silenced genes.

In the field of cancer biology, there is growing appreciation that abnormalities in histone modification and DNA methylation pathways can drive tumorigenesis across many cancer types and there is promise for improved therapies aimed at reversal of gene silencing [20]. In this study, we bridge the study of the Xi with the development of strategies to more efficiently demethylate and reactivate silenced genes. 5-aza-2′-dC is used clinically in the setting of hematologic malignancies with the rationale of reactivating silenced genes [19]. The drug is currently approved for the treatment of myelodysplastic syndrome (MDS) and acute myeloid leukemia (AML) [20]. Several studies have confirmed that 5-aza-2′-dC at low doses elicits genome-wide DNA demethylation in AML patient samples [21–23]. One approach to increase the epigenetic activity of 5-aza-2′-dC in myeloid malignancy is to use it in combination with other agents known to elicit reactivation of silenced genes, such as histone deacetylase inhibitors [20]. Notably, for the Xi, such co-treatment approaches increase the rate of XCR in cell culture systems [9]. The similar efficacy of 5-aza-2′-dC alone or in combination with other chromatin-modifying agents in Xi-linked genes and in myeloid leukemia supports the translation of findings from X-chromosome inactivation to epigenetic cancer therapies.

Here, we set out to find additional pathways that in combination with 5-aza-2′-dC, elicit XCR. Specifically, we applied high-throughput siRNA and chemical screening to identify factors that could reactivate a silent reporter transgene that is specifically located on the Xi. Our screen employed treatment with a low dose of 5-aza-2′-dC to sensitize somatic cells for DNA demethylation and XCR, which on its own is not sufficient to induce these effects. We identified that inhibition of the ribonucleotide reductase protein complex significantly enhances the DNA demethylation action of 5-aza-2′-dC and hence the activity of the Xi-reporter. We characterize the mechanism of action as increasing DNA incorporation of 5-aza-2′-dC and thus its demethylating activity. While our approach initially centered on the Xi, we found a pathway that altered DNA methylation levels genome-wide. Our study therefore demonstrates that assays of XCR can be adapted to optimize the epigenetic activity of a DNA demethylating drug combination.

## Results and discussion

### An siRNA screen for XCR in the presence of a low 5-aza-2′-dC dose identifies the ribonucleotide reductase pathway

**Fig. 1:**
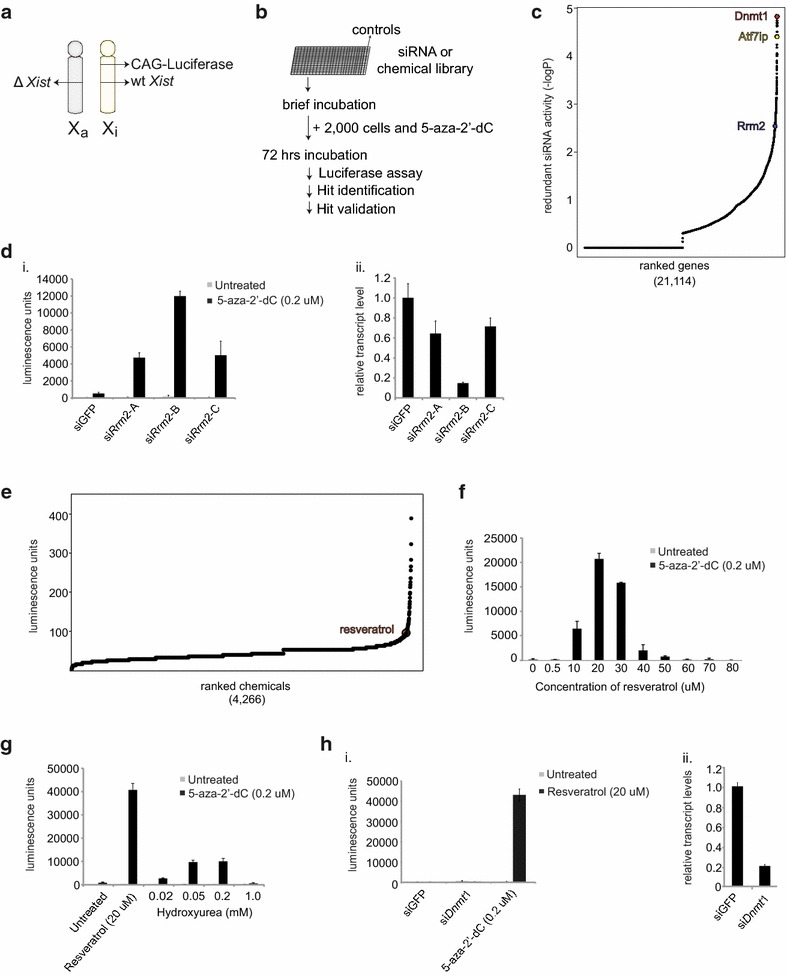
High-throughput siRNA and chemical screens identify RRM2 depletion and resveratrol as mediators of XCR. **a** Schematic of the X chromosomes in female reporter MEFs carrying the luciferase reporter transgene in the *Hprt* locus specifically on the Xi. The *Xist* deletion on one of the chromosomes skews X-inactivation to the wild-type *Xist*-bearing X chromosome. **b** Diagram of the screening workflow. siRNAs from the mouse genome-wide library and selected chemical libraries were assayed in 384-well plates containing a column of positive and negative controls. Xi-luciferase reporter MEFs were added and incubated for 72 h in the presence of 5-aza-2′-dC (0.2 μM) prior to a luciferase assay. **c** Gene activity distribution plot ranked by the –log of the p-value obtained with the redundant siRNA activity (RSA) assay from duplicate genome-wide siRNA screens following transformation of the luminescence activity values into robust z-scores. The top validated hits, *Dnmt1*, *Atf7ip,* and *Rrm2,* are labeled. **d** (*i*) Graph depicting Xi-luciferase reporter reactivation upon knockdown of *Rrm2* with the three siRNAs (A, B, C) obtained from the genome-wide library in the presence or absence of 5-aza-2′-dC (0.2 μM) in the 12-well format. Luminescence was measured 72 h after the start of the treatment. *Error bars* indicate standard deviation of luminescence unit values from three individual wells with a given treatment in one experiment. (*ii*) RT-qPCR for RNA levels of *Rrm2* normalized to si*GFP* control and *Gapdh* expression. RNA was harvested in parallel to luciferase assays shown in (*i*). *Error bars* indicate standard deviation from three measurements in one experiment. **e** Activity of chemicals in the chemical screen in the presence of 5-aza-2′-dC (0.2 μM), ranked by luminescence unit with the value corresponding to resveratrol designated. **f** Xi-luciferase reporter assay as described in (di) titrating the resveratrol concentration with or without 5-aza-2′-dC (0.2 μM). **g** Xi-luciferase reporter assay as in (di) titrating hydroxyurea (HU) with or without (untreated) 5-aza-2′-dC (0.2 μM). The result for resveratrol treatment in the same experiment is given for comparison. **h** (*i*) Xi-luciferase reporter assay as in (di) comparing the consequences of 0.2 μM 5-aza-2′-dC treatment and siRNA-mediated knockdown of *Dnmt*1 to elicit reporter reactivation by 20 μM resveratrol. (*ii*) RT-qPCR for *Dnmt1* RNA levels normalized to si*GFP* control and *Gapdh* expression in the same experiment as (*i*)

Previous work from our lab has shown that an Xi-linked, CAG promoter-driven luciferase transgene in the *Hprt* locus (Xi-luciferase) is a sensitive reporter of gene silencing on the Xi when tested in primary mouse embryonic fibroblasts (MEFs) [24]. Our Xi-luciferase MEFs faithfully inactivate the luciferase-bearing X chromosome in female embryonic development rather than undergoing random XCI because an *Xist* deletion on the other X chromosome forces XCI on the chromosome carrying the wild-type *Xist* allele [25] (Fig. 1a). The Xi-luciferase gene body and promoter are highly methylated at the DNA level and Xi-luciferase reporter MEFs increase luciferase activity in a dose-dependent fashion in response to 5-aza-2′-dC treatment [24]. Here, we used Xi-luciferase reporter MEFs to screen for gene knockdowns or chemicals that could elicit XCR.

In order to perform a high-throughput screen for XCR, we established an siRNA knockdown assay in 384-well format, with each individual siRNA tested in a single well. As positive control, we chose knockdown of *Dnmt1* since interference with *Dnmt1* either by knockout or 5-aza-2′-dC treatment has previously been described to elicit XCR in MEFs [9, 24]. Initially, we tested increasing concentrations of 5-aza-2′-dC in combination with *Dnmt1* knockdown to determine a 5-aza-2′-dC concentration for which the depletion of *Dnmt1* by siRNAs yielded robust reporter reactivation, but where 5-aza-2′-dC treatment alone does not elicit reactivation. Our titration experiment demonstrated that the combination of 5-aza-2′-dC at a concentration ranging from 0.1 to 0.2 μM, along with si*Dnmt1* treatment, enhanced luciferase activity in the 384-well format. Importantly, 5-aza-2′-dC treatment in this concentration range or knockdown of *Dnmt1* alone did not induce a significant difference in luciferase signal compared to untreated wells (Additional file 1: Figure S1A). The requirement for 5-aza-2′-dC co-treatment with *Dnmt1* knockdown to detect XCR likely reflects that *Dnmt1* knockdown alone does not lead to sufficient levels of XCR detected in the small 384-well format assay. By comparison, a higher dose of 5-aza-2′-dC (1 μM) elicited strong reactivation of the Xi-linked luciferase reporter on its own that was not as dramatically enhanced by si*Dnmt1* treatment (Additional file 1: Figure S1A). Thus, a low dose of 5-aza-2′-dC has a sensitizing effect on eliciting XCR by *Dnmt1* knockdown. The interaction of 5-aza-2′-dC with other Xi maintenance factors indicates a similar sensitizing effect with respect to XCR. For instance, the knockdown of the candidate Xi-maintenance factor *Atf7ip* or deletion of *Xist* produces a low rate of XCR that is significantly boosted by the addition of 5-aza-2′-dC [9, 24]. Therefore, we extended the low concentration 5-aza-2′-dC treatment (0.2 μM) to the entire genome-wide siRNA screen with the rationale that knockdown of other chromatin-modifying factors may require concurrent DNA demethylation to produce strong Xi-luciferase reporter reactivation.

We performed a genome-wide mouse siRNA screen with 51,150 siRNAs against 21,114 genes on 153 384-well plates (see “Methods” section for details on the library used) in duplicate using female Xi-linked luciferase reporter MEFs in the presence of low 5-aza-2′-dC. We measured luciferase levels 72 h after siRNA transfection (Fig. 1b). To eliminate batch effects, we normalized luminescence data by 384-well plate, and then analyzed the data by prioritizing gene hits with multiple active siRNAs by redundant siRNA activity (RSA) analysis [26] (Fig. 1c, Additional file 2: Figure S2). Notably, *Dnmt1* was the top hit in our genome-wide screen, which provided internal validation of the method (Fig. 1c). Further support came from another hit, identified as *Atf7ip*, which our group recently reported as a maintenance factor in XCI [24]. As with other previously described maintenance factors, we found that the Xi-luciferase signal in response to knockdown of *Atf7ip* was greatly increased by low 5-aza-2′-dC (0.2 μM) co-treatment [24]. Identification of *Atf7ip* in the screen supports the strategy of 5-aza-2′-dC co-treatment to unmask functional contribution of Xi-maintenance factors.

To select novel hits, we chose the top 54 genes from the RSA analysis with at least two unique active siRNAs inducing an increase in luciferase levels in the 384-well screen, omitting genes we deemed irrelevant such as those for olfactory receptors, and retested the active siRNAs sequences from the library (Additional file 3: Table S1). Several of these siRNAs showed reproducible increases in luciferase activity in the validation assay (Additional file 4: Figure S3). We decided to focus on *Rrm2* as a hit since one siRNA against it had produced the next highest level of luciferase activity in the validation assay after the siRNAs targeting *Atf7ip* or *Dnmt1*. Follow-up assays with a greater number of starting cells demonstrated an increase in luciferase activity for each of our three different siRNAs against the ribonucleotide reductase (RNR) M2 subunit gene (*Rrm2*) (Fig. 1d). The luminescence generated with si*Rrm2* treatment was in proportion to individual extent of *Rrm2* knockdown, suggesting specificity of *Rrm2* targeting for the XCR effect (Fig. 1d).

As part of the RNR enzyme complex, RRM2 catalyzes the conversion of ribonucleoside 5′-disphosphates to their 2′-deoxyribonucleoside form in the rate-limiting step of de novo dNTP biosynthesis [27]. The RRM2 subunit, which was identified in this siRNA screen, is specifically upregulated at S phase of cell cycle and is necessary for the activity of the RNR complex [27]. Since we identified si*Rrm2* in combination with 5-aza-2′-dC (0.2 μM) in the genome-wide screen, we next asked if knockdown of *Rrm2* could also elicit XCR in the absence of 5-aza-2′-dC, since interference with *Atf7ip* or *Xist* produces a low rate of XCR that is significantly boosted by the addition of 5-aza-2′-dC [9, 24]. However, unlike previously described Xi-maintenance factors, we did not find that si*Rrm2* produced luciferase activity in the absence of 5-aza-2′-dC (Fig. 1d). These results were reproduced with an Xi-linked fluorescent reporter (Additional file 5: Figure S4B/C) [9]. We conclude that low doses of 5-aza-2′-dC are necessary for the XCR effect of si*Rrm2* identified by our genome-wide screen for factors involved in the maintenance of Xi silencing.

### Chemical inhibitors of ribonucleotide reductase elicit XCR in the presence of 5-aza-2′-dC

We used a complimentary approach to further probe the pathways contributing to Xi maintenance by performing a companion screen analogous to the siRNA screen but instead using a collection of annotated chemicals (Fig. 1b, Additional file 6: Figure S5A). In the screen, we found that resveratrol, a chemical agent known for mimicking cellular effects of caloric restriction, demonstrated the potential to activate the Xi-luciferase reporter (Fig. 1e) [28]. To validate the screening result, we tested the effect of various resveratrol concentrations on the Xi-luciferase reporter. The bell-shaped dose–response activity of resveratrol in combination with fixed, low concentration of 5-aza-2′-dC (0.2 μM) indicated a maximal XCR activity at a concentration of 20 μM (Fig. 1f). In order to confirm an XCR-specific effect, we tested whether the combination of resveratrol with 5-aza-2′-dC could reactivate different Xi-linked reporters. We found that 20 μM resveratrol and 5-aza-2′-dC (0.2 μM) together could also reactivate two fluorescent reporters including the CAG-driven H2B citrine transgene within the *Hprt* locus and a distal CAG-driven GFP transgene (Additional file 6: Figure S4) [9, 24]. As previously observed, the proportion of cells expressing the reporter differs for Xi-CAG-H2B-citrine and Xi-GFP MEFs likely owing to different silencing requirements of the two loci on the Xi [24].

Resveratrol is a naturally-occurring polyphenolic molecule believed to have numerous direct intracellular protein targets [29]. It is described to mediate its metabolic effects through direct and indirect activation of the histone deacetylase SIRT1 though no specific role in reversal of chromatin silencing or effects on the Xi has been described [30–32]. Of note, we did not find that knockdown of *Sirt1* attenuated the ability of resveratrol with 5-aza-2′-dC to elicit Xi-luciferase reactivation (not shown). A further search for the cellular target of resveratrol in XCR led us to a study that described resveratrol as an inhibitor of RNR, the same enzyme complex that we identified as a hit in the genome-wide siRNA screen for XCR described above [33]. This link between our complimentary screening approaches pointed to resveratrol’s role in XCR in the presence of 5-aza-2′-dC by means of RNR inhibition.

In order to further investigate whether RNR is the target of resveratrol in eliciting XCR, we tested a well-characterized inhibitor of RNR, hydroxyurea (HU), and found that it also increased Xi-luciferase activity in the presence of a low dose of 5-aza-2′-dC (0.2 μM) (Fig. 1g). From the titration, HU had a maximum effect on Xi-luciferase reactivation at 50 and at 200 μM and a fading effect at 20 μM (Fig. 1g). HU was not detected from the chemical library because it was assayed at a screening concentration of 10 μM, which was probably insufficient concentration to detect activity. 50 μM HU treatment in combination with low 5-aza-2′-dC also induced reactivation of the Xi-linked GFP (Additional file 5: Figure S4B/C). We reasoned that if resveratrol and HU converge on inhibition of RNR, that the XCR effect of resveratrol and HU should require the co-treatment with 5-aza-2′-dC as seen for the *Rrm2* knockdown. Indeed, we found that similar to the si*Rrm2* condition, resveratrol and HU treatment demonstrated a complete dependence on low levels of 5-aza-2′-dC to elicit XCR (Fig. 1d/f/g, Additional file 5: Figure S4B/C). We conclude that RNR inhibition alone by these various means does not increase Xi-luciferase activity.

Since the Xi-luciferase reporter assay is not reflective of cell number, we measured protein concentration in luciferase assay lysates to rule out variable cell number due to different treatments as an explanation for lack of Xi-reporter activation in the absence of 5-aza-2′-dC (Additional file 7: Figure S6A). Variations in protein lysate concentration were minor across all treatments, indicating RNR inhibition requires the presence of 5-aza-2′-dC to elicit its effect on XCR. The identification of chemicals with RNR-inhibiting activity added support to the XCR role of inhibiting this pathway in the presence of 5-aza-2′-dC.

We further investigated the relationship between 5-aza-2′-dC and RNR inhibition in XCR by querying whether 5-aza-2′-dC can be replaced by knockdown of *Dnmt1*. In previous studies where 5-aza-2′-dC had a sensitizing effect towards XCR, the effect is attributable to interference with *Dnmt1* [24]. For instance, 5-aza-2′-dC treatment could be substituted by knockdown of *Dnmt1* to elicit synergistic XCR by *Atf7ip* knockdown [24]. Contrary to these prior findings, we found that *Dnmt1* depletion by siRNAs did not replace the contribution of 5-aza-2′-dC to XCR induced by RNR-inhibition via resveratrol (Fig. 1h). Together, these findings suggest a mechanism of action whereby RNR inhibition specifically affects the action of cytidine analog 5-aza-2′-dC.

### RNR inhibition increases incorporation of 5-aza-2′-dC into DNA

**Fig. 2:**
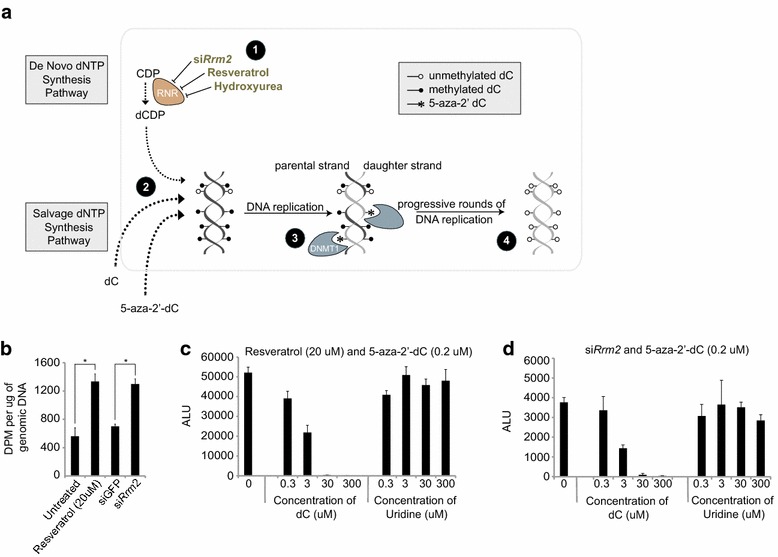
Inhibition of RNR enhances DNA incorporation of 5-aza-2′-dC to elicit XCR. **a** Illustration of model in which (*1*) inhibition of ribonucleotide reductase (RNR) by various means leads to (*2*) increased relative dCTP utilization for DNA synthesis from salvage pathways which are supplemented with exogenous 5-aza-2′-dC. (*3*) DNMT1 inhibition occurs upon binding to DNA-incorporated 5-aza-2′-dC leading to (*4*) increased loss of DNA methylation with successive cell divisions. **b** Quantification of 3H-5-aza-2′-dC (3H-Decitabine) incorporation into genomic DNA upon either resveratrol treatment or *Rrm2* knockdown for 48 h. Genomic DNA was isolated and an equal volume measured for 3H-Decitabine incorporation (disintegrations per minute, DPM), then normalized to the amount of DNA loaded (μg). *Error bars* indicate standard deviation from three independent treatment wells. Asterisks indicate p < 0.01 by Student’s T-test. **c** Xi-luciferase reactivation assay as in Fig. 1di in the presence of 0.2 μM 5-aza-2′-dC and 20 μM resveratrol and increasing concentrations of deoxycytidine (dC) or uridine. ALU represents luminescence unit values. **d** As in **c** except with si*Rrm2* in the place of resveratrol

Next, we sought to understand how RRM2 inhibition interacts with low amounts of 5-aza-2′-dC to elicit XCR. The pool of dNTPs in the nucleus is tightly regulated and studies have speculated that RNR inhibition can increase the likelihood of nucleoside analog DNA incorporation by reducing the pools of endogenous nucleotide concentrations [27, 34]. Accordingly, we postulated that RRM2 inhibition may increase 5-aza-2′-dCTP concentration in the nucleus relative to the endogenous dCTP pool, leading to more 5-aza-2′-dCTP DNA incorporation (Fig. 2a). Higher rates of 5-aza-2′-dC incorporation into DNA subsequently could lead to greater DNA demethylation and XCR (Fig. 2a).

Consistent with this model, we observed that knockdown of *Rrm2* or resveratrol treatment reproducibly increased the amount of tritiated 5-aza-2′-dC incorporated into DNA approximately by two-fold (Fig. 2b). We further tested the role of the ratio of 5-aza-2′-dCTP to endogenous dCTP by the converse manipulation of increasing dCTP relative to 5-aza-2′-dC. This experiment was performed by adding increasing concentrations of deoxycytidine (dC) into media, which is metabolized to dCTP within the cell, in the presence of 5-aza-2′-dC with resveratrol treatment (Fig. 2c) or *Rrm2* knockdown (Fig. 2d). Importantly, dC does not require the action of RNR for DNA incorporation. Our expectation was that an increase in dCTP levels in the cell would reduce the incorporation of 5-aza-2′-dC into the DNA, and therefore reduce the reactivation of the Xi-linked luciferase reporter. As expected, the luciferase signal decreased in a dose-dependent fashion when exogenous deoxycytidine was supplied in the media (Fig. 2c/d). The loss of the Xi-reporter reactivation is consistent with the notion that the relative nuclear concentration of 5-aza-2′-dCTP to dCTP is shifted by the addition of an exogenous nucleotide substrate to reduce the effective concentration of the 5-aza-2′-dC analog (Fig. 2c/d).

An alternate explanation for the observed decrease in luciferase signal upon addition of dC is a reduction in cell number. To rule out possible nucleotide treatment-dependent cell growth effects, we confirmed that protein concentrations in lysates were similar for the various treatment conditions (Additional file 7: Figure S6B/C). Furthermore, we used uridine as a control because it is a nontoxic precursor of pyrimidine synthesis that, like deoxycytidine, can be taken up by cells and used as a substrate via the nucleoside salvage synthetic pathway (Fig. 2c/d) [35]. However, unlike deoxycytidine, uridine requires reduction by RNR in order to contribute to dNTP pools [35]. Increasing levels of uridine did not alter Xi-luciferase levels and thereby XCR in the presence of 5-aza-2′-dC with *Rrm2* knockdown and resveratrol treatment, respectively, compared to control (Fig. 2c/d, Additional file 7: Figure S6B/C). These results support the role of deoxycytidine in reversing the XCR effect downstream of RNR.

In summary, RRM2/RNR inhibition was identified in the XCR screen because it augmented 5-aza-2′-dC DNA incorporation. This mechanism is consistent with the observation that RNR inhibition alone, i.e. in the absence of 5-aza-2′-dC, did not produce measurable Xi-reporter reactivation in prior assays.

### RRM2 inhibition enhances genome-wide demethylation caused by 5-aza-2′-dC in MEFs

**Fig. 3:**
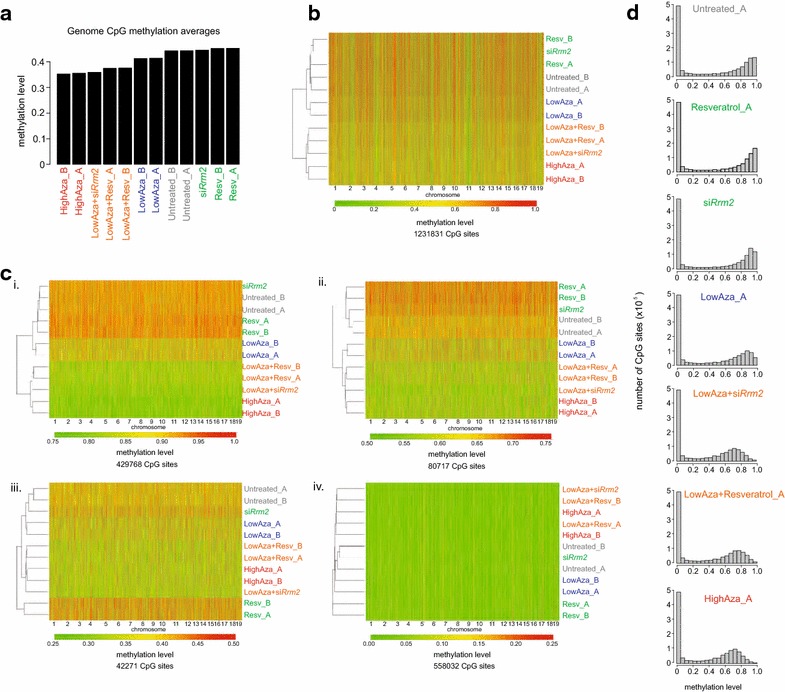
RNR inhibition increases 5-aza-2′-dC-mediated genome-wide DNA demethylation in MEFs. **a** Bar chart displaying average genome-wide CpG methylation levels for the indicated 72 h treatments filtered for CpGs with at least 5X sequencing coverage by RRBS across all samples. Label color reflects the various treatment groups. *Subscripts* (A and B) indicate replicates where applicable. Treatment concentrations are: LowAza (5-aza-2′-dC 0.2 μM), HighAza (5-aza-2′-dC 10.0 μM), and Resv (resveratrol 20 μM). **b** Heat map of unsupervised hierarchical clustering of DNA methylation levels for all autosomal CpGs assayed by RRBS in MEFs treated with the indicated chemicals for 72 h as in **a** with at least 5X sequencing coverage across all samples. A methylation level of 1 indicates 100 % methylation, while 0 represents complete absence of methylation. **c** Heat maps as in **b** but for subsets of autosomal CpG sites partitioned into four groups representing different DNA methylation levels in the untreated control samples; (*i*) 0.75–1.0, (*ii*) 0.50–0.75, (*iii*) 0.25–0.50, and (*iv*) 0–0.25. In each case, the combination of RNR inhibition with 0.2 μM 5-aza-2′-dC clusters away from all other samples, but together with the high dose of 5-aza-2′-dC, and is more demethylated. **d** Histograms display DNA methylation distributions for all autosomal CpGs as in **b** for indicated treatments and replicates. Data for additional replicates can be found in Additional file 8: Figure S7C/D

If RRM2 inhibition potentiates low dose 5-aza-2′-dC action to increase XCR by increasing the incorporation of 5-aza-2′-dC, then DNA methylation levels in cells treated with a low dose of 5-aza-2′-dC with RRM2 inhibition should approximate those of cells treated with a high dose of 5-aza-2′-dC. We investigated DNA methylation patterns at genome-scale by reduced representation bisulfite sequencing (RRBS) [36]. Specifically, MEFs were treated with si*Rrm2* or resveratrol alone, low or high doses of 5-aza-2′-dC, and combinations of si*Rrm2* or resveratrol with a low dose of 5-aza-2′-dC (Fig. 3, Additional file 8: Figure S7, Additional file 9: Figure S8).

As expected, based on global methylation averages, hierarchical clustering, and methylation distributions, the treatment of MEFs with a low dose of 5-aza-2′-dC (0.2 μM) induced a smaller reduction in the level of genome-wide methylation than the high dose of 5-aza-2′-dC (10.0 μM), which resulted in marked demethylation compared to control samples (Fig. 3a, b, d, Additional file 8: Figure S7C/D). We found that treatment with si*Rrm2* or resveratrol alone (without 5-aza-2′-dC) marginally increased global DNA methylation levels compared to untreated samples (Fig. 3a, b, d, Additional file 8: Figure S7C/D). Notably, the combination of *Rrm2* knockdown or resveratrol with the low dose 5-aza-2′-dC reduced global methylation to a similar extent as the high dose 5-aza-2′-dC treatment (Fig. 3a, b, d, Additional file 8: Figure S7C/D). These effects on the methylation profile were similar for autosomes and the X chromosome at the global scale (Fig. 3b, Additional file 9: Figure S8A) as well as on promoters and CpG islands (Additional file 8: Fig. S7A, Additional file 9: Figure S8B). These findings are consistent with a genome-wide effect on DNA methylation rather than an Xi-specific mechanism, owing to increased DNA incorporation of 5-aza-2′-dC under RRM2 inhibition conditions. We observed that CpGs with the highest levels of methylation in the control samples showed the most dramatic 5-aza-2′-dC-induced demethylation (Fig. 3c, Additional file 8: Figure S7B). For CpGs with lower methylation levels in the untreated conditions, demethylation due to 5-aza-2′-dC incorporation is still visible but less extensive (Fig. 3c, Additional file 8: Figure S7B). We believe that the greater apparent effect in highly methylated regions does not represent a predilection of 5-aza-2′-dC for highly methylated regions, as has been previously suggested [21], but rather that the random incorporation of 5-aza-2′-dC disproportionately affects the methylation estimates of highly methylated sites.

We also extracted the available methylation data for the Xi-linked luciferase reporter gene to determine whether the methylation levels correlated with the extent of Xi-luciferase reactivation in the various conditions. We found that CpG sites within the luciferase reporter gene followed the genome-wide methylation changes, and that the low 5-aza-2′-dC treatment together with RNR inhibition, by either *Rrm2* knockdown or resveratrol, induced similar demethylation as the high dose of 5-aza-2′-dC (Additional file 10: Figure S9). The concordant behavior of Xi-luciferase reporter CpG sites supports the conclusion that the augmentation of DNA incorporation of 5-aza-2′-dC describes the *Rrm2* result in our Xi-reporter reactivation screen.

Taken together, our genome-wide methylation analysis for low dose 5-aza-2′-dC with RRM2 inhibition supports the idea that RRM2 inhibition increases the effective concentration of 5-aza-2′-dC and thereby its DNA incorporation, leading to global DNA demethylation.

### Hydroxyurea and 5-aza-2′-dC synergistically inhibit myeloid leukemia cell line proliferation in a dose-dependent fashion

**Fig. 4:**
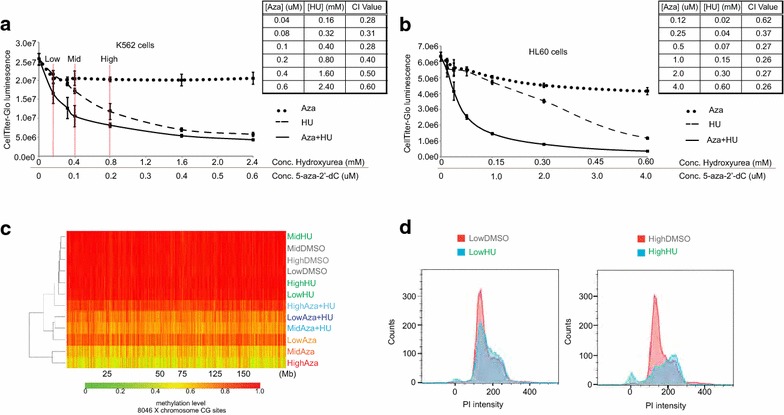
Hydroxyurea and 5-aza-2′-dC treatment of myeloid leukemia cell lines. **a** Dose–response curves measuring viable K562 cells using the Cell-Titer Glow Assay. Chemical treatments were performed with HU or 5-aza-2′-dC (Aza) alone or in combination at a fixed concentration ratio of 4000:1 HU:Aza (based on individual IC50 values). *Red lines* indicate concentrations assayed in the subsequent RRBS analysis displayed in **c**. The accompanying table depicts the Chow-Talalay analysis of the Combination Index (CI) at the given treatment combination concentrations. CI values <1.0 indicates synergy, 1= additive effect, and >1.0 antagonism of the combination drug effect. **b** As in **a** except dose–response curves for HL60 cells using a fixed concentration ratio of 150:1 HU:Aza in the combined treatments. **c** Heat map showing an unsupervised hierarchical clustering of X chromosome CpG methylation levels in K562 cells treated with the indicated chemicals for 72 h. Only those CpGs with greater than 0.75 methylation level in the DMSO-treated samples are displayed, as these are the CpGs most dramatically affected by 5-aza-2′-dC treatment (see Fig. 3). CpGs were filtered for at least 10× sequencing coverage across all samples. DNA methylation levels were profiled for the treatments with the HU/aza concentration combinations indicated with *red lines* in **a** (labeled Low, Mid, High). In addition, we treated cells with either 5-aza-2′-dC or HU at the respective concentrations (Low HU (0.16 mM), Mid HU (0.4 mM), High HU (0.80 mM), and Low Aza (0.04 μM), Mid Aza (0.1 μM), High Aza (0.2 μM)). Aza was resuspended in DMSO, thus DMSO only controls are matched to volume of Aza added in corresponding Aza samples. **d** Flow cytometry analysis of propidium iodide-stained K562 cells treated with low and high HU concentrations as described in **c** compared to low or high DMSO control treatment

Given that RRM2 inhibition increases DNA incorporation of 5-aza-2′-dC, we next applied the combination of RRM2 inhibition and 5-aza-2′-dC to a disease model in which 5-aza-2′-dC has therapeutic relevance. 5-aza-2′-dC is an FDA-approved drug and commonly used off-label in the setting of acute myeloid leukemia (AML) [20]. Therefore, we tested the drug combination in four myeloid leukemia cell lines (THP1, U937, K562, HL60) (Fig. 4, Additional file 11: Figure S10). We hypothesized that, since RRM2 inhibition increased DNA incorporation of 5-aza-2′-dC, the combination of RRM2 inhibition with 5-aza-2′-dC could improve the therapeutic index of 5-aza-2′-dC, allowing lower doses to maximize demethylation activity with fewer cytotoxic off-target effects. We chose to use HU as the form of RRM2 inhibition because it also is an FDA-approved agent commonly used off-label for cyto-reductive purposes, also in the setting of AML [37].

To assess the effect of combining HU and 5-aza-2′-dC on myeloid leukemia cell line proliferation, we applied a luminescence-based cell viability assay that linearly scales with cell number and titered HU and 5-aza-2′-dC individually to determine IC50 values for each cell line (Additional file 11: Figure S10A). For 5-aza-2′-dC, IC50 values were difficult to approximate given a plateau in cell proliferation changes at higher concentrations (not shown). Thus we chose the 5-aza-2′-dC concentration corresponding to halfway to the point of plateau effect. We then combined HU and 5-aza-2′-dC at fixed ratios, empirically determined for each of the four myeloid leukemia cell lines (Fig. 4a/b, Additional file 11: Figure S10B/C). In each of the four cell lines tested, the combination treatment inhibited cell proliferation more than either treatment alone. In order to make a quantitative determination of the drug interaction, we calculated Chou-Talalay Combination Indices (CI) where CI <1, =1, >1 indicate synergism, additive effect, and antagonism, respectively [38]. The combination of HU and 5-aza-2′-dC demonstrated evidence of drug synergism across a range of fixed drug concentration ratios in the four cell lines tested (Fig. 4a, b, Additional file 11: Figure S10B/C). We repeated the drug treatments with K562 cells in a soft agar assay and confirmed the synergistic effect of HU and 5-aza-2′-dC on clonal cell expansion (Additional file 11: Figure S10D). Consistent with the proliferation studies, the combination HU and 5-aza-2′-dC reduced colony formation to a greater extent compared to either treatment alone. Together, these results demonstrate a synergistic interaction between HU and 5-aza-2′-dC in the control of cell proliferation.

We next assessed whether DNA demethylation related to the synergistic drug effect observed. Specifically, we determined the DNA methylation profile of K562 cells treated at a low, mid, and high concentration of 5-aza-2′-dC and HU at a fixed ratio by RRBS (Fig. 4c, Additional file 12: Figure S11). The low average genome-wide CpG methylation levels of approximately 35 % in K562 cells with few highly methylated CpGs is consistent with a prior studies reporting overall global hypomethylation inherent to K562 cells (Additional file 12: Figure S11, DMSO-treated control conditions) [39]. Nonetheless, treatment with a fixed ratio of low HU and 5-aza-2′-dC concentrations, that induced a synergistic effect on cell growth (Fig. 4a, low condition), reduced DNA methylation compared to the low 5-aza-2′-dC treatment alone (Fig. 4c). As expected, HU treatment alone did not alter DNA methylation levels (Fig. 4c). As with MEFs, filtering by CpGs that are highly methylated in control conditions best displayed the enhancing effect of low HU to low 5-aza-2′-dC concentrations (Fig. 4c, Additional file 12: Figure S11A).

Unexpectedly, methylation levels did not appreciably decrease and even increased with the higher dose combinations of HU and 5-aza-2′-dC (mid and high treatment combinations) (Fig. 4c). Particularly at the high concentration combination, HU addition almost completely blunted the effect of 5-aza-2′-dC on methylation (Fig. 4c, Additional file 12: Figure S11). We hypothesized that the differing effects of the low and high concentration combinations may be due to interference of cell cycle progression with increasing concentrations of HU, which in turn interferes with the incorporation of 5-aza-2′-dC into DNA during DNA replication. Accordingly, flow cytometry analysis revealed a significant cell-cycle arrest of K562 cells at the high HU concentration, but not at the low concentration (Fig. 4d).

Our data suggest that at lower concentrations, HU and 5-aza-2′-dC act synergistically on cell growth, at least partially via DNA demethylation, while at higher concentrations, direct effects on cell cycle progression inhibit cell growth not allowing DNA demethylation via 5-aza-2′-dC, which is replication dependent. Regardless, these data indicate that the combination of HU and 5-aza-2′-dC synergistically decreases cell proliferation of the four myeloid leukemia cell lines tested. Moreover, the mechanism of action of this synergistic drug combination changes in a dose-dependent fashion.

## Conclusions

Using an Xi-linked luciferase reporter sensitized to reactivate by low concentration 5-aza-2′-dC treatment, we screened genome-wide siRNA and chemical libraries for reactivation activity. We found that inhibition of the RRM2 subunit of the ribonucleotide reductase enzyme increases rates of Xi-linked reporter reactivation. We attribute the effect of RRM2 inhibition on the Xi in MEFs to augmentation of 5-aza-2′-dC incorporation into DNA, which in turn induces increased genome-wide DNA demethylation in a pattern similar to a high dose 5-aza-2′-dC treatment alone. Moreover, treatment of myeloid leukemia cells with 5-aza-2′-dC and the RRM2-inhibitor HU together synergistically inhibited cell proliferation and altered DNA methylation levels in these cancer cell lines in a dose-dependent manner. These findings suggest RRM2-inhibitors improve the demethlyation activity of 5-aza-2′-dC and may have clinical benefit if used in combination.

Our screen utilized a single copy Xi-linked reporter to identify the effect of RRM2 inhibition, which was then characterized as a genome-wide effect of augmenting 5-aza-2′-dC-mediated demethylation. The extension of our findings from a single gene reporter on the Xi to a genome-wide effect indicates that the Xi can be used as a model system for identifying and targeting general mechanisms of gene silencing. The optimal dose-schedule of 5-aza-2′-dC remains to be determined and the most effective epigenetic therapy will likely require use of 5-aza-2′ dC in combination with other epigenetic agents [40]. The XCR assay may be helpful to accomplish these objectives. The robust nature of Xi silencing in differentiated cells, however, contributes to one of the challenges of high-throughput screening with this model: XCR is partial and occurs at low rates, thus XCR assays must be optimized in sensitivity. Previous Xi maintenance screens have used pooled shRNA libraries in combination with immortalized Xi-GFP transgene-bearing reporter fibroblasts [41, 42]. The list of Xi-maintenance candidate factors from these prior studies is distinct from ours, with the exception of *Dnmt1*, for several potential reasons. First, previous approaches did not screen in the presence of 5-aza-2′-dC and are thus not expected to find 5-aza-2′-dC-augmenting pathways such as RRM2-inhibition. Second, cell line immortalization has the potential to create aberrancies in chromatin silencing pathways that deviate from normal development, as in cancer. Therefore, using primary MEFs, as in our screen, may more closely reflect in vivo silencing contributions of Xi maintenance pathways. However, our single-well format using individual siRNAs presents challenges in detecting rare Xi reactivation events, even if adapted to a more sensitive luciferase reporter gene. Our screen was likely underpowered to identify novel high-confidence Xi silencing pathways as reflected by a low signal-to-noise margin of the assay, expressed as a low Z-factor of 0.11 (Additional file 1: Figure S1B). Improvement of the assay using different co-treatments (besides 5-aza-2′-dC) to increase rates of XCR, may lead to identification of different classes of Xi maintenance factors and minimize screening false negatives and positives [43].

Regardless, the adoption of 5-aza-2′-dC in the optimization of this screen in order to sensitize for DNA demethylation ultimately led to identification of a 5-aza-2′-dC-interacting pathway with therapeutic relevance. From the standpoint of optimizing epigenetically acting drugs, monitoring gene reactivation from the Xi can therefore provide a readout of chromatin reprogramming with immediate effects on gene expression.

We used cell proliferation assays and genome-wide methylation level estimates in myeloid leukemia cell lines to gauge the activity of 5-aza-2′-dC. Our data suggest that at a low concentration of 5-aza-2′-dC, the addition of low dose HU, increases the fraction of 5-aza-2′-dC that is incorporated into DNA and available to inhibit DNMT1. This DNA incorporation augmentation effect has the potential to represent a therapeutic advantage. RRM2-inhibitors such as resveratrol and hydroxyurea improve the demethylation activity of 5-aza-2′-dC and may have clinical benefit if used in combination. The clinical use of 5-aza-2′-dC is hampered by incomplete disease response in AML and MDS and by high rates of adverse effects [18, 44, 45]. Its mechanism of action in patients is most likely due to a combination of demethylating and direct cytotoxic actions that differ in their relative contribution according to disease context and 5-aza-2′-dC concentration. At higher doses, 5-aza-2′-dC is thought to form DNA adducts leading to DNA synthesis arrest, which inhibits its DNA incorporation [20, 46]. Higher doses therefore contribute to higher rates of adverse reactions including hematologic toxicities [20]. Accordingly, lower doses have been favored in more recent clinical trials and have shown greater likelihood in eliciting gene expression changes as well as producing clinic responses in AML and even solid tumors [20, 22, 46]. Thus, increasing DNA incorporation of 5-aza-2′-dC at low doses is a promising strategy to increase its therapeutic index by biasing its activity profile towards DNA demethylation.

In this study, we observed synergistic an anti-proliferative effect of 5-aza-2′-dC in combination with HU, however, did not capture genome-wide methylation changes at all concentrations to explain this effect. The anti-proliferative effect in the absence of global DNA methylation changes is likely secondary to cytotoxic effects such as DNA adduct formation and DNA synthesis arrest. Alternatively, it is possible that differentially methylated loci are preferentially demethylated by 5-aza-2′-dC at lower concentrations and expression of these genes drives the phenotypic effects of inhibiting proliferation, even when mean global methylation levels are not affected.

Previous studies have reported that 5-aza-2′-dC and HU drug combination is antagonistic to DNA methylation based on bisulfite sequencing analysis of three loci in two other cancer cell lines [47]. Our data support these findings at high concentration HU with 5-aza-2′-dC in K562 cells but shows a synergistic effect on DNA demethylation at lower doses of HU and with RNR inhibition. The extent of RNR inhibition is likely critical for a synergistic interaction with 5-aza-2′-dC as too little RNR inhibition will not increase DNA incorporation of 5-aza-2′-dC and too much RNR inhibition with lead to S-phase arrest and interfere with 5-aza-2′-dC-mediated passive DNA demethylation (see model Fig. 2a).

Another relevant disease model to test a potential therapeutic benefit of the combination of 5-aza-2′-dC and HU may be sickle cell anemia. Current therapies to treat the genetic defect in adult hemoglobin are aimed at reactivating the fetal hemoglobin gene [48]. Hydroxyurea is a standard therapy that when administered at cytotoxic doses to patients severely affected with sickle cell anemia increases fetal hemoglobin levels, but only in a subset of patients for unknown reasons [48]. As opposed to myeloid leukemia, where the efficacy of 5-aza-2′dC is partially attributable to demethylation, in sickle-cell anemia clinical responses to 5-aza-2′-dC do correlate with demethylation of the fetal hemoglobin gene and increases in hemoglobin levels [48–50]. Thus it is appealing to explore modified dosing schedules of HU and 5-aza-2′-dC for sickle cell patients already receiving these therapies in order to potentially exploit some synergistic effect of combination therapy for raising hemoglobin levels.

## Methods

### Genome-wide siRNA library plate preparation

The Silencer Mouse Druggable siRNA Library V3 and Extension set V3 (Ambion) were provided as 250 pmol of lyophilized powder in a total of 153 384-well source plates, containing one siRNA per well except in columns 23 and 24, which were reserved for controls. Each of 21,114 genes is represented by mostly 3 unique (some 2 unique) siRNAs on different 384-well plates. Plates were centrifuged at 1700×*g*, 50 μl of nuclease-free water was added to each well, sealed and briefly vortexed to resuspend the siRNAs in individual wells. RNA concentrations were confirmed by measuring 1 μl of siRNA solution from 14 randomly chosen wells by NanoDrop spectrophotometer (Thermo Scientific). 2 μl of siRNA diluted to 0.5 pmol/μl from each source plate was stamped in duplicate onto Matrix white opaque 384-well tissue culture-treated plates (Thermo Scientific) by BenchCel 4X system with a PlateLoc plate sealer, Vcode Barcode Printer, and Vprep pipette fitted with a 96 LT head (all from Agilent Technologies) and stored in −80°.

### Derivation of MEFs

Xi reporter MEFs were derived from a cross between transgenic male mice bearing a CAG promoter-driven luciferase, H2B-Citrine allele in the *Hprt* locus, and the X-linked GFP, respectively, and transgenic female mice heterozygous for an *Xist* knockout allele [24]. MEFs were derived at embryonic day 14.5 and cultured in MEF media (DMEM supplemented with 10 % FBS, nonessential amino acids, l-glutamine, penicillin–streptomycin, β-mercaptoethanol) following standard procedures. The reporter MEFs with genotypes Xi^CAG−Luciferase^Xa^ΔXist^, Xi^CAG−H2BCitrine^Xa^ΔXist^, and Xi^GFP^Xa^ΔXist^ were obtained at expected Mendelian ratios of 1 out of 4 embryos and identified by PCR genotyping for presence of an *Xist* knockout allele, presence of a FLP-Frt recombination production in the *Hprt* locus and GFP, respectively, and lack of Y chromosome gene *Zfy* [24].

### High-throughput screening siRNA and chemical screening assays

The screening assay was optimized to maximize the Z-factor statistical measure of signal-to-noise ratio between the positive control of *Dnmt1* knockdown and negative control or no siRNA mock-transfected cells [51]. Pilot experiments sequentially tested individual variables of the assay such as incubation times and reagent types to increase the Z-factor of the assay. The 5-aza-2′-dC concentration of 0.2 uM used in the screen was determined in this empiric fashion, by titrating a range of 5-aza-2′-dC concentrations to determine which would maximally increase the signal separation between *Dnmt1* knockdown and control samples, calculated as the Z-factor of the assay. The Z-factor of the finalized screening assay was 0.11 (Additional file 1: Figure S1B) [51]. Screening data analysis was performed by first normalizing raw luminescence values by robust z-score which is the number of median absolute deviations for a given well luminescence value from the plate median luminescence value [52].

Primary MEFs from four female Xi-luciferase reporter embryos were thawed in 15 cm^2^ plates, passaged twice at a 1:6 split, pooled to ensure a homogeneous cell population, and then frozen into 144 vials for use in screening and hit validation. For the large-scale screen, for each batch of 30 plates carrying the genome-wide siRNA library, 2 vials of cells were thawed in MEF media. After 1 day in culture, adherent cells were trypsinized, live cells excluding Trypan blue were counted using a hemocytometer and brought up in suspension with MEF media agitated by a stir bar.

Meanwhile, a batch of 30 plates including duplicates from 15 source plates of 384-well siRNA library were thawed at room temperature, centrifuged, and cleaned with RNAse-reducing solution (Life Technologies). A positive control siRNA targeting *Dnmt1* (Ambion AM161526) was stamped by BenchCel 4X system with an 8 channel LT head (Agilent Technologies) into 16 wells of column 24 of each library plate by adding 4ul of nuclease-free water containing 1 pmol of si*Dnmt1* to each well. The 16 wells of the column 23 were reserved as negative control and contained no siRNA. Transfection was initiated by adding 20 μl of Opti-MEM (Life Technologies) and 0.05 μl RNAimax (Life Technologies) per well by Multidrop 384 (Thermo Scientific) and incubating for 20 min to 1 h. 20 ul of cell suspension containing 2000 cells with 5-aza-2′-dC (0.4 μM, Sigma) was added to the transfection mix, bringing the final 5-aza-2′-dC concentration to 0.2 μM. Cells were incubated for 3 days in a humidified 37° incubator at 5 % CO_2_. 20 μl of media was then aspirated off using an ELx 405 plate washer (BioTek Instruments) and 20 μl of One-Glo luciferase assay reagent (Promega) was added using the Multidrop 384 and incubated for 20 min. As luminescence data were collected on an Acquest reader (Molecular Devices), quality control for each plate was performed by visual inspection of positive and negative controls on the heat map during data collection.

Chemical screening was performed analogously with several exceptions: 384-well plates were not pre-treated. Rather, 50 μl of cell suspension with 2000 MEFs and 5-aza-2′-dC (0.2 μM) were plated in fifteen 384-well plates. A positive control mixture was distributed to a row of wells on each plate by mixing 50 μl of cell suspension with 2000 cells per well in 1× MEF media with high concentration 5-aza-2′-dC (10.0 μM). The screening compounds were added to all but positive control wells as 0.5 μl of 1 mM stock in DMSO by Biomek FX (Beckman Coulter). After 72 h incubation, 30 μl of media were aspirated off, and the luciferase assay was performed as described for the siRNA screen. Libraries screened include 4266 compounds from Microsource (2000), Biomol enzyme inhibitor (337) and bioactive lipid libraries (203), Prestwick chemical library (1120), and NIH clinical collections (606) at the UCLA MSSR [53]. The 30 chemicals producing highest luciferase values were chosen for subsequent validation.

### High-throughput siRNA screening analysis

Genome-wide siRNA screen hits were identified by Redundant siRNA Activity (RSA) analysis using robust z-scores as the input values [26]. The R script provided by Konig et al. was used with minor modifications to adapt it for our workflow (http://carrier.gnf.org/publications/RSA). RSA works by ranking hits in order of activity then assigning P values for genes based on whether their siRNAs rank higher than would be expected by chance. We obtained two activity measurements for each siRNA since the siRNA library was screened in duplicate, and treated these data points as independent measurements with regard to the analysis. Therefore, most genes were represented by six data points (and some with four data points) in the RSA analysis.

### Cell culture and treatment methods

For subsequent Xi-reactivation/validation assays, MEFs at passage 1 or 2 post-derivation were seeded at a density of 6.0 × 10^4^ cells per 12-well well and chemicals in MEF media and/or siRNAs in Opti-MEM media (Gibco) were added and incubated for 72 h. For 5-aza-2′-dC (Sigma), which was resuspended in DMSO and stored at −80 °C, final DMSO concentration on the cells was kept below 0.1 %. Total volumes of MEF and/or Opti-MEM media were normalized across samples when different treatments were used. Hydroxyurea and resveratrol (Sigma) were resuspended in DMSO and Uridine and Deoxycytidine (Sigma) were resuspended in water and stored at −20 °C. K562, HL60, U937, and THP1 cells were purchased from ATCC. K562, U937, and THP1 cells were cultured in RPMI media (Gibco) with 10 % FBS and HL60 cells were cultured in IMDM (Gibco) with 20 % FBS. ATCC culture method suggestions were followed for expanding the cells. The soft agar assay was performed by mixing of 1.2 % nobel agar (Sigma) in water with 2X RPMI to achieve final concentration of 0.6 % agar for the bottom layer. After this solidified in 6-well plates, top soft agar was prepared at final 0.3 % nobel agar concentration containing K562 cells to achieve 4.0 × 10^4^ cells per well. DMSO or 5-aza-2′dC (0.05 μM) and/or HU (0.05 mM) were added to both bottom and top agar layers. This 1000:1 ratio of HU to 5-aza-2′-dC was determined to be optimal for the soft agar assay, which is different from the 4000:1 optimal ratio used in CellTiter Glo assay. Small colonies started appearing 4 days after plating. On day 8, colonies were stained with 0.01 % crystal violet for 1 h, washed with PBS, and the plates were scanned to obtain images.

### Luciferase assay

For each luciferase assay, MEF Xi-luciferase reporter treatments were performed in triplicate 12-well wells for 72 h and lysed with 200 μl passive lysis buffer (PLB, Promega) for 20 min at room temperature on an orbital shaker. Lysates were cleared by 30 s of centrifugation and 20 μl were assayed for luciferase activity with 50 μl of LARI reagent (Promega) on a GloMax microplate luminometer (Promega). Protein concentration measurements were performed on corresponding PLB lysates by Quick Start™ Bradford Protein Assay Kit (Bio-Rad) and analyzed by interpolating to standard curve according to the manufacturer’s instruction. For the proliferation assays of leukemia cell lines, 100 μl of well-suspended cells were mixed with 100 μl of CellTiter Glo^®^ reagent (Promega), incubated at room temperature for 20 min, and luciferase units were measured using a GloMax microplate luminometer (Promega).

### RT-qPCR analysis

Cells were harvested from a 6-well format in TRIzol (Invitrogen) and RNA purification was performed with the RNeasy kit (Qiagen) according to manufacturer’s instructions with on-column DNAse treatment. cDNA was prepared using SuperScript III (Invitrogen) with random hexamers and RT-qPCR was performed using a M×3000 thermocycler (Stratagene) with primers for *Rrm2* (F-GCACTGGGAAGCTCTGAAAC, R-GGCAATTTGGAAGCCATAGA), *Dnmt1* (F-CATGAATTCCTGCAAACAGAA, R-TTGACTTTAGCCAGGTAGCC), or *Gapdh* (F- GGCCTTCCGTGTTCCT, R-GCCTGCTTCACCACCTTCT). Results were normalized to *Gapdh* by the ΔCt method.

### Knockdowns in follow-up experiments

Knockdowns by siRNA were performed by reverse transfection at 25 nM final concentration of siRNA. Briefly, a cell suspension was added to a pre-incubated mixture of Lipofectamine RNAimax, 100 μl of reduced serum Opti-MEM media, and siRNA. The siRNAs used were *Rrm2* [Ambion, 150659 (A), 64497 (B), 150661 (C)], *Dnmt1* (Ambion, 161526), and, as negative controls, *Scramble* (Ambion, 4636), *Luciferase* (Dharmacon, D-001210-02), *Aurkb* (Dharmacon, D-063793-01), and *GFP* (Dharmacon, P-002048-01). For *Rrm2* knockdown where the siRNA is not specified, siRNA 66497 was used.

### Flow cytometry

Flow cytometry for measuring the reactivation of the Xi-linked H2B Citrine and Xi-GFP reporters was performed as described previously [24]. For the cell cycle measurement with K526 cells, 5.0 × 10^6^ cells (determined by trypan blue exclusion assay) were taken from each treatment condition, washed once with PBS, and stained with propidium iodide buffer (3 mM EDTA pH 8.0, 0.05 % NP40, 50 μg/ml PI, 1 mg/ml RNaseA in PBS) for 30 min at room temperature. Stained cells were passed through a strainer and analyzed by FACSDiva (BD Biosciences) with FlowJo software (Tree Star, Inc.).

### 3H decitabine incorporation

This assay was analogous to the reactivation treatment assays with a few modifications: assays were scaled 2.5-fold to 6-well format, 1 μl (1 μCi) of tritiated 5-aza-2′-dC (3H-Decitabine, Moravek Biochemicals Inc.) was added instead of cold 5-aza-2′-dC, and samples were harvested after 48 h of incubation. Cells were trypsinized, genomic DNA isolated using the Quick-gDNA MinPrep kit (Zymo Research), and measured by QuBit fluorometer (Life Technologies). Tritium content of 25 μl of genomic DNA was measured using a scintillation counter and normalized to the measured DNA concentration.

### Reduced representation bisulfite sequencing

Primary Xi-reporter MEFs were subjected to the same chemical treatment as used for the luciferase assays, but in 6-well format, and a fraction of the cells was taken to confirm appropriate luciferase reporter activity. Genomic DNA was isolated using the Blood and Cell Culture Mini Kit (Qiagen) with RNase A treatment (Life Technologies). The RRBS libaries were generated at previously described by Orozco et al. with minor modifications [54]. DNA purifications for each enzymatic reaction was carried out using AMPure XP beads (Beckman Coulter). Bisulfite conversion was performed using the Epitect kit (Qiagen) twice compared to manufacturer’s instruction to optimize the efficiency. Bisulfite-converted libraries were amplified using MyTaq Mix (Bioline) with the following program: (98 °C for 15 s, 60 °C for 30 s, 72 °C for 30 s) 12 cycles, 72 °C for 5 min, 4 °C storage. DNA Methylation calling was performed using BS-Seeker2 (2.0.32) using Bowtie (0.12.9) for read alignment on the UCLA Hoffman2 computer cluster [55]. Reads with adapter contamination were trimmed. The adapter sequence used for the contamination check was as follows: CGAGATCGGAAGAGCACACGTC, i.e. meCG end repair ± A tail ± 10 bp of Illumina adapter sequence. CpG islands (CGIs) were obtained from UCSC (http://genome.ucsc.edu) and CGI tracks were based on methods by Gardiner-Garner and Frommer [56]. Promoters were defined as the region transcription start site (TSS) minus 1 kb to TSS for all UCSC genes. Only sites covered by at least five reads across all samples under consideration were used in an effort to obtain reliable methylation levels. The methylation levels of samples were hierarchically clustered using complete linkage and the Euclidean distance metric. Statistical analysis, clustering, and heat map generation were performed using custom R scripts [57] (R core team, http://www.r-project.org).

### Public availability of data

All genome-wide data are available from the GEO resource at http://www.ncbi.nlm.nih.gov/geo/query/acc.cgi?acc=GSE72295.

## Additional files

10.1186/s13072-015-0034-4 Optimization of 5-aza-2’-dC concentration for the genome-wide siRNA screen.** A.** Bar chart illustrating luciferase activity from Xi-reporter MEFs upon knockdown of *Dnmt1* and treatment with varying concentrations of 5-aza-2’-dC in 384-well format for 72 hours. Error bars indicate standard deviation from eight measurements in one experiment. Aterisks indicate p < 0.01 by Student’s T-test. **B.** Scatterplot of luminescence values from the optimized Xi-reactivation screening assay in 384-well format in the presence of 5-aza-2’-dC (0.2 μM) with si*Dnmt1* (red) or negative control si*Aurkb* (Aurora kinase B, blue). The Z-factor, a measure of separation between positive and negative control populations used in the assessment of high-throughput assays, is shown [52].
10.1186/s13072-015-0034-4 Batch effects of genome-wide siRNA screening and robust z-score normalization.** A.** Box plot of all raw luciferase measurements distributions per individual 384-well plate from one of the duplicates of the siRNA screen. These plates were prepared and assayed in 30-plate batches according to their numerical order in the source library plates, keeping duplicate plates together. **B.** As in (A) except each measurement was normalized by the robust z-score (median absolute deviations from the plate median [52].
10.1186/s13072-015-0034-4 Redundant siRNA activity ranking of genome-wide siRNA screen data. Table is color-coded to reflect which hit genes were validated.
10.1186/s13072-015-0034-4 Validation of gene hits identified by genome-wide siRNA screening. The chart displays the luminescence for the Xi-luciferase assay in 24-well format with knockdown by the indicated siRNAs, chosen as top hits of the genome-wide screen, in combination with 5-aza-2’-dC (0.2 μM) for 72 hours. For each gene hit, siRNAs were re-ordered to match the sequences of the 2 or 3 active siRNA identified by RSA activity analysis of the genome-wide siRNA screen. Error bars indicate one standard deviation from duplicate wells. si*Dnmt1* positive control is shown in red.
10.1186/s13072-015-0034-4 Validation of the resveratrol result with different Xi-reporter lines.** A.** Diagram of MEF Xi-H2B Citrine reporter genotype. As in Fig. 1A, except the Xi is bearing a CAG-driven histone H2B-Citrine reporter gene instead of luciferase in the *Hprt* locus The chart summarizes flow cytometry analysis of Xi-H2B Citrine reporter MEFs treated with resveratrol (20 μM) and/or 5-aza-2’-dC (0.2 μM or 10 μM) for 72 hours.** B.** Diagram of MEF Xi-GFP reporter genotype. The Xi is bearing a randomly integrated CAG-driven GFP allele near the centromere [58]. The chart summarizes flow cytometry analysis of GFP reporter MEFs treated with siRrm2, resveratrol (20 uM) or HU (0.05 mM), and DMSO or 5-aza-2’-dC (0.2 μM). Error bars represent standard deviation from triplicate wells.** C.** Representative flow cytometry dot plots of GFP reporter MEFs from part B.
10.1186/s13072-015-0034-4 Chemical screen results and validation.** A.** Box plot of all raw luciferase measurements from the chemical screen by individual 384-well plate, demonstrating lack of obvious batch effect. Chemical library plates were prepared and assayed as one batch of 15 plates. **B.** Chart displaying results from the Xi-luciferase assay in the 24-well format upon treatment with various chemicals (at 10 μM) in the presence of 5-aza-2’-dC (0.2 μM) for 72 hours. Error bars indicate one standard deviation from duplicate wells except for negative control 5-aza-2’-dC (0.2 μM) alone (n=16) and positive control 5-aza-2’-dC (10.0 μM) alone (n=16). Resveratrol is indicated with an asterisk.
10.1186/s13072-015-0034-4 Protein concentration measurements for Xi-luciferase reactivation assays. **A.** Chart depicts protein concentration of cell lysates corresponding to luciferase measurements in (1G). Error bars indicate standard deviation from three individual wells. **B.** As in (A) but protein concentrations of cell lysates corresponding to luciferase measurements for (2C). **C.** As in (A) but protein concentrations of cell lysates corresponding to luciferase measurements for (2D).
10.1186/s13072-015-0034-4 Analysis of autosomal DNA methylation in MEFs treated with combinations of RNR inhibition and 5-aza-2’-dC. **A.** (i) Heat map of the unsupervised hierarchical clustering as in Fig. 3B but only for autosomal CpG sites within CpG islands (CGIs). Genomic locations of CpG islands were obtained from UCSC Genome browser (see "Methods" section). Constitutively hypermethylated (>0.75) and hypomethylated (<0.15) sites were filtered out to improve contrast. (ii) Heat map of the unsupervised hierarchical clustering as in Fig. 3B but only for autosomal CpG sites within promoters. As in (i), constitutively hypermethylated (>0.75) and hypomethylated (<0.15) sites were filtered out to improve contrast. Promoters were defined as the region 1 kb upstream of the TSS for all UCSC genes. **B.** Heat maps of the unsupervised hierarchical clustering as in Fig. 3C for autosomal CpGs within CGIs with at least 5X coverage by RRBS across samples, but filtered for sites with methylation levels in the untreated sample of either (i) 0.75–1.0 (ii) 0.50–.75 (iii) 0.25–0.50 or (iv) 0–0.25. **C.** As in Fig. 3D but for replicate samples. **D.** Pairwise significance test results conducted using a two-sample Kolmogorov-Smirnov test (‘KS-test stat’ and ‘KS-test p-value’ columns) between the distributions of autosomal CpG methylation in Fig. 3D and Figure S7C, as well as two measures of effect size: Cohen’s *d* and the differences between these ‘upper modes’ between the comparisons (‘Delta upper mode’ column).
10.1186/s13072-015-0034-4 Analysis of DNA methylation status on the X chromosome in MEFs treated with combinations of RNR inhibition and 5-aza-2’-dC. **A.** Heat map of the unsupervised hierarchical clustering of CpG methylation levels in MEFs as in Fig. 3B, except that the data for X chromosome CpG sites are shown. **B.** (i) Heat map of the unsupervised hierarchical clustering as in Fig. 3B but only for CpG sites within CpG islands on the X chromosome. Constitutively hypermethylated (>0.75) and hypomethylated (<0.15) sites were filtered out to improve contrast. (ii) As in (i) except for CpG sites within promoters on the X chromosome. Again, constitutively hypermethylated (>0.75) and hypomethylated (<0.15) sites were filtered out to improve contrast. Promoters were defined as the region 1 kb upstream of the TSS for all UCSC genes.
10.1186/s13072-015-0034-4 DNA methylation status of the luciferase transgene in MEFs treated with combinations of RNR inhibition and 5-aza-2’-dC. **A.** Bar chart displaying average CpG methylation levels for the indicated treatments filtered by CpGs with at least 5X sequencing coverage by RRBS across all samples as in Fig. 3A, but only considering the CpGs in the luciferase reporter gene/promoter. **B.** Heat map of unsupervised hierarchical clustering of CpG methylation levels as in Fig. 3B except for CpG sites in the luciferase reporter gene/promoter. **C.** As in (B), except for CpG sites within the luciferase reporter with a methylation level greater than 0.75 in both of the untreated samples. **D.** Histograms showing the distribution of CpG methylation levels within the luciferase reporter gene.
10.1186/s13072-015-0034-4 Synergistic effect of HU and 5-aza-2’dC on myeloid leukemia cell line proliferation.** A.** Graphs represent cell counts measured with the hemocytometer after trypan blue staining compared to viable cell number measurement determined by CellTiter-Glo reagent (Promega) for four myeloid leukemia cell lines. High correlation coefficient, R^2^, demonstrates linear relationship. **B.** Dose response curves as in Fig. 4A, except for THP1 cells using a fixed concentration ratio of 1000:1 HU:Aza. **C.** Dose response curves as in Fig. 4A, except for U937 cells using a fixed concentration ratio of 300:1 HU:Aza. **D.** Soft agar assay of K562 cells plated in DMSO or 5-aza-2’dC (0.05 uM) and/or HU (0.05 mM) in a final concentration of 3% agar and stained with crystal violet after 8 days of growth.
10.1186/s13072-015-0034-4 Extended data on the methylation analysis of K562 cells. **A.** Heat map showing an unsupervised hierarchical clustering of X chromosome CpG methylation in K562 cells treated with the indicated chemicals for 72 hours as in Fig. 4C but for all X chromosome CpGs. **B.** CpG methylation distribution along the X chromosome in K562 cells, for CpG sites with at least 10X coverage across all samples as determined by RRBS. Chemical treatments are as shown in Fig. 4A/C.

## Abbreviations

XCI: X chromosome inactivation
XCR: X chromosome reactivation
Xi: inactive X chromosome
MEFs: mouse embryonic fibroblasts
RNR: ribonucleotide reductase
HU: hydroxyurea
