# Supplementary figures and images for: A high-throughput screen of inactive X chromosome reactivation identifies the enhancement of DNA demethylation by 5-aza-2′-dC upon inhibition of ribonucleotide reductase
Source: Epigenetics Chromatin. 2015 Oct 13;8:42. doi: 10.1186/s13072-015-0034-4 (PMC4604769; doi:10.1186/s13072-015-0034-4)

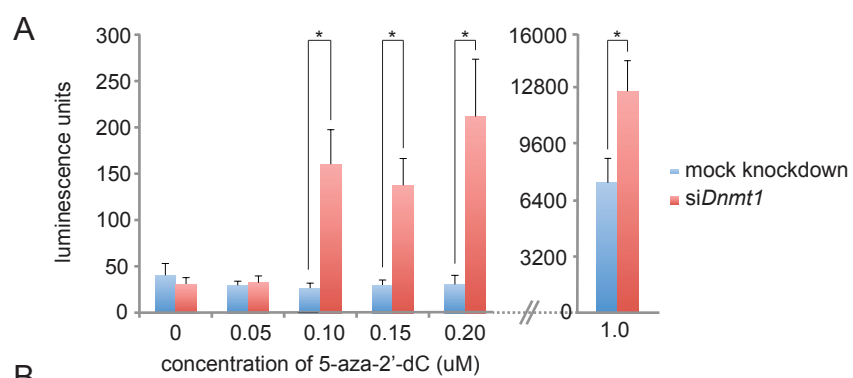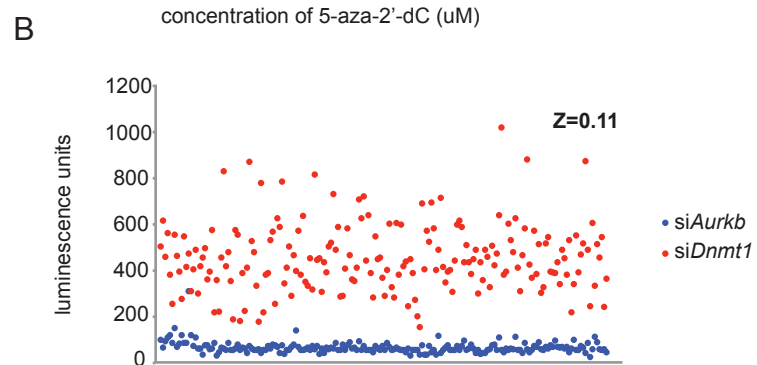

Supplement: Supplementary file 1 — 10.1186/s13072-015-0034-4 Optimization of 5-aza-2’-dC concentration for the genome-wide siRNA screen. A. Bar chart illustrating luciferase activity from Xi-reporter MEFs upon knockdown of Dnmt1 and treatment with varying concentrations of 5-aza-2’-dC in 384-well format for 72 hours. Error bars indicate standard deviation from eight measurements in one experiment. Aterisks indicate p < 0.01 by Student’s T-test. B. Scatterplot of luminescence values from the optimized Xi-reactivation screening assay in 384-well format in the presence of 5-aza-2’-dC (0.2 μM) with siDnmt1 (red) or negative control siAurkb (Aurora kinase B, blue). The Z-factor, a measure of separation between positive and negative control populations used in the assessment of high-throughput assays, is shown [52]. [file 13072_2015_34_MOESM1_ESM.pdf]

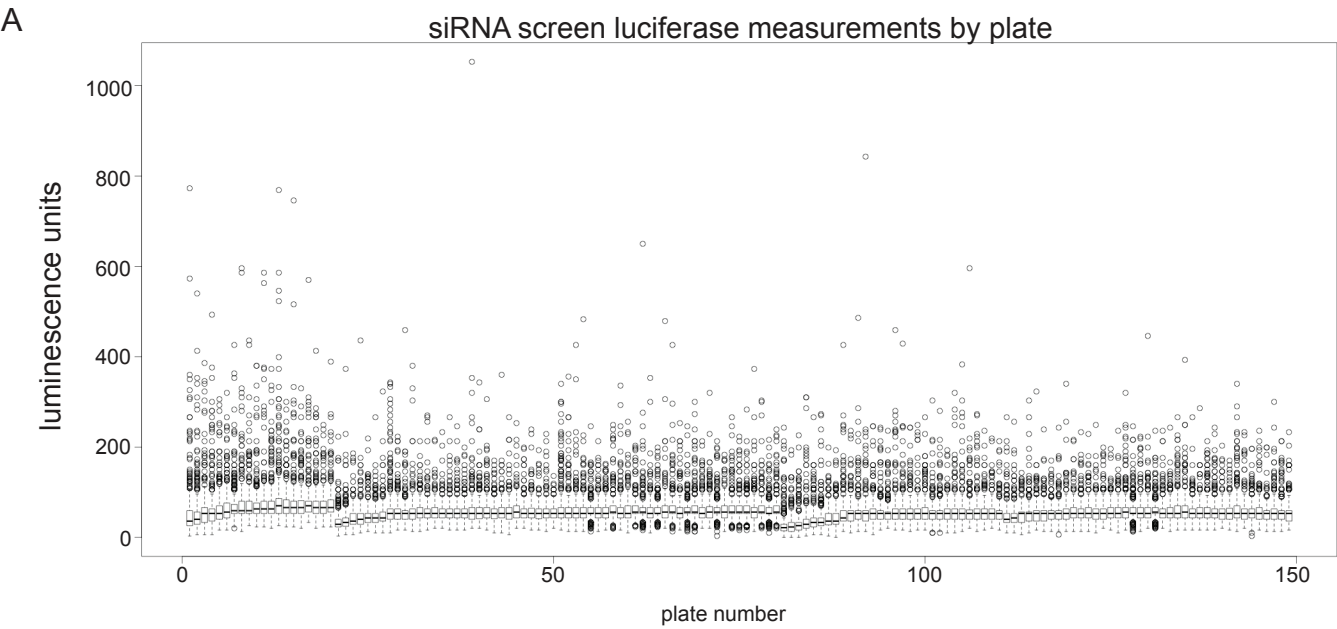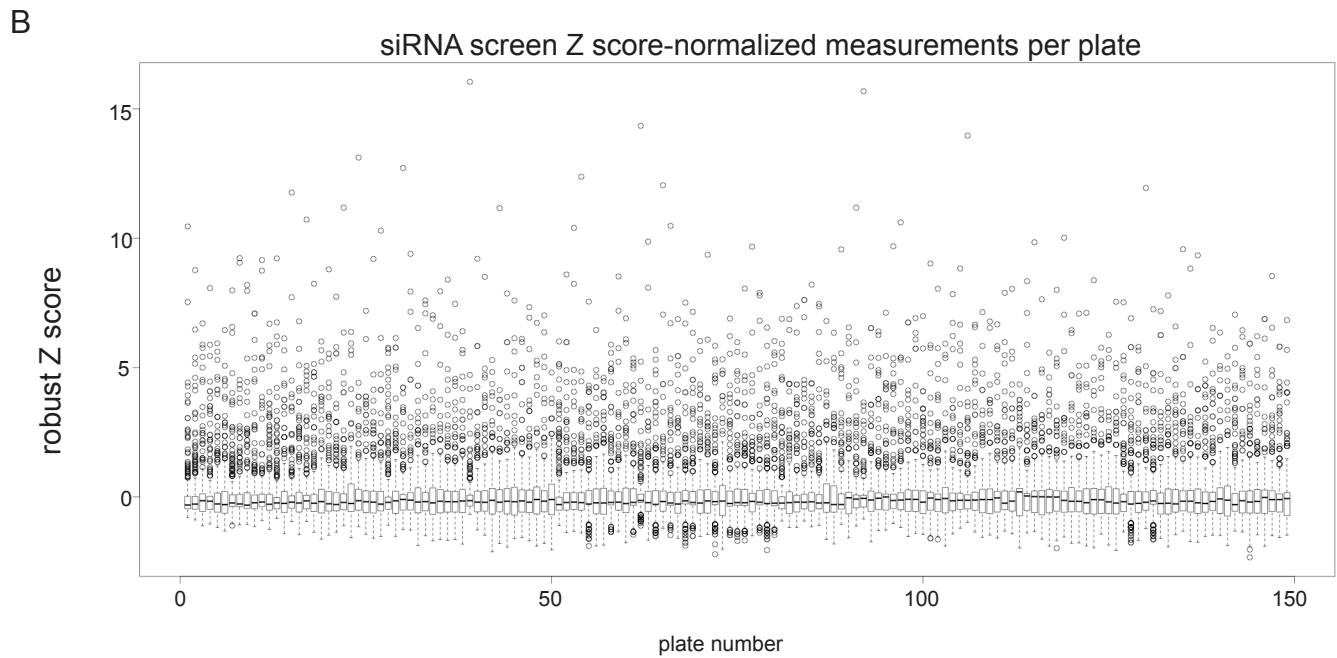

Supplement: Supplementary file 2 — 10.1186/s13072-015-0034-4 Batch effects of genome-wide siRNA screening and robust z-score normalization. A. Box plot of all raw luciferase measurements distributions per individual 384-well plate from one of the duplicates of the siRNA screen. These plates were prepared and assayed in 30-plate batches according to their numerical order in the source library plates, keeping duplicate plates together. B. As in (A) except each measurement was normalized by the robust z-score (median absolute deviations from the plate median [52]. [file 13072_2015_34_MOESM2_ESM.pdf]

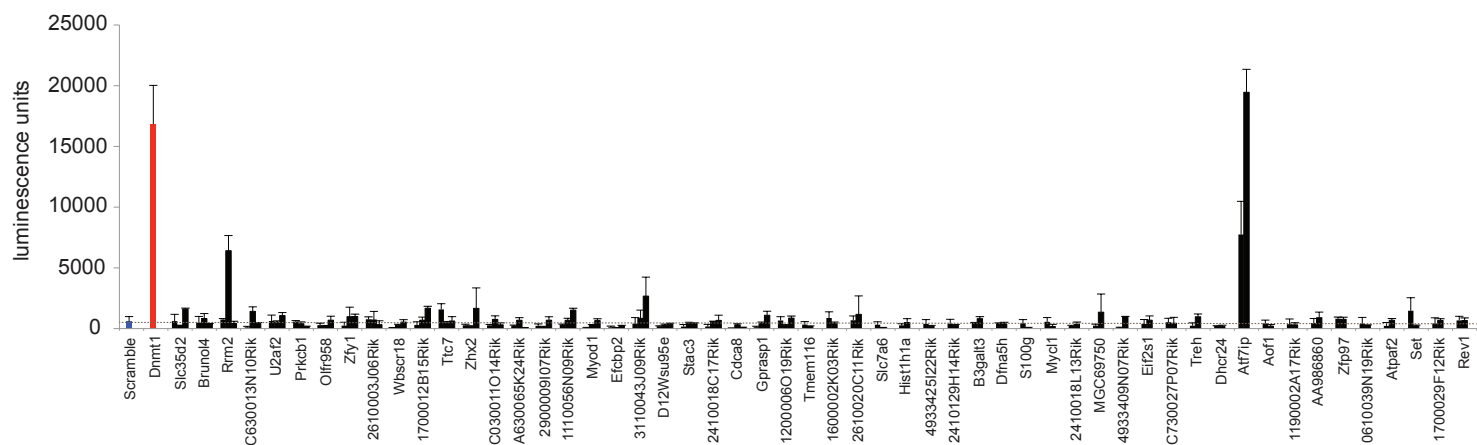

Supplement: Supplementary file 4 — 10.1186/s13072-015-0034-4 Validation of gene hits identified by genome-wide siRNA screening. The chart displays the luminescence for the Xi-luciferase assay in 24-well format with knockdown by the indicated siRNAs, chosen as top hits of the genome-wide screen, in combination with 5-aza-2’-dC (0.2 μM) for 72 hours. For each gene hit, siRNAs were re-ordered to match the sequences of the 2 or 3 active siRNA identified by RSA activity analysis of the genome-wide siRNA screen. Error bars indicate one standard deviation from duplicate wells. siDnmt1 positive control is shown in red. [file 13072_2015_34_MOESM4_ESM.pdf]

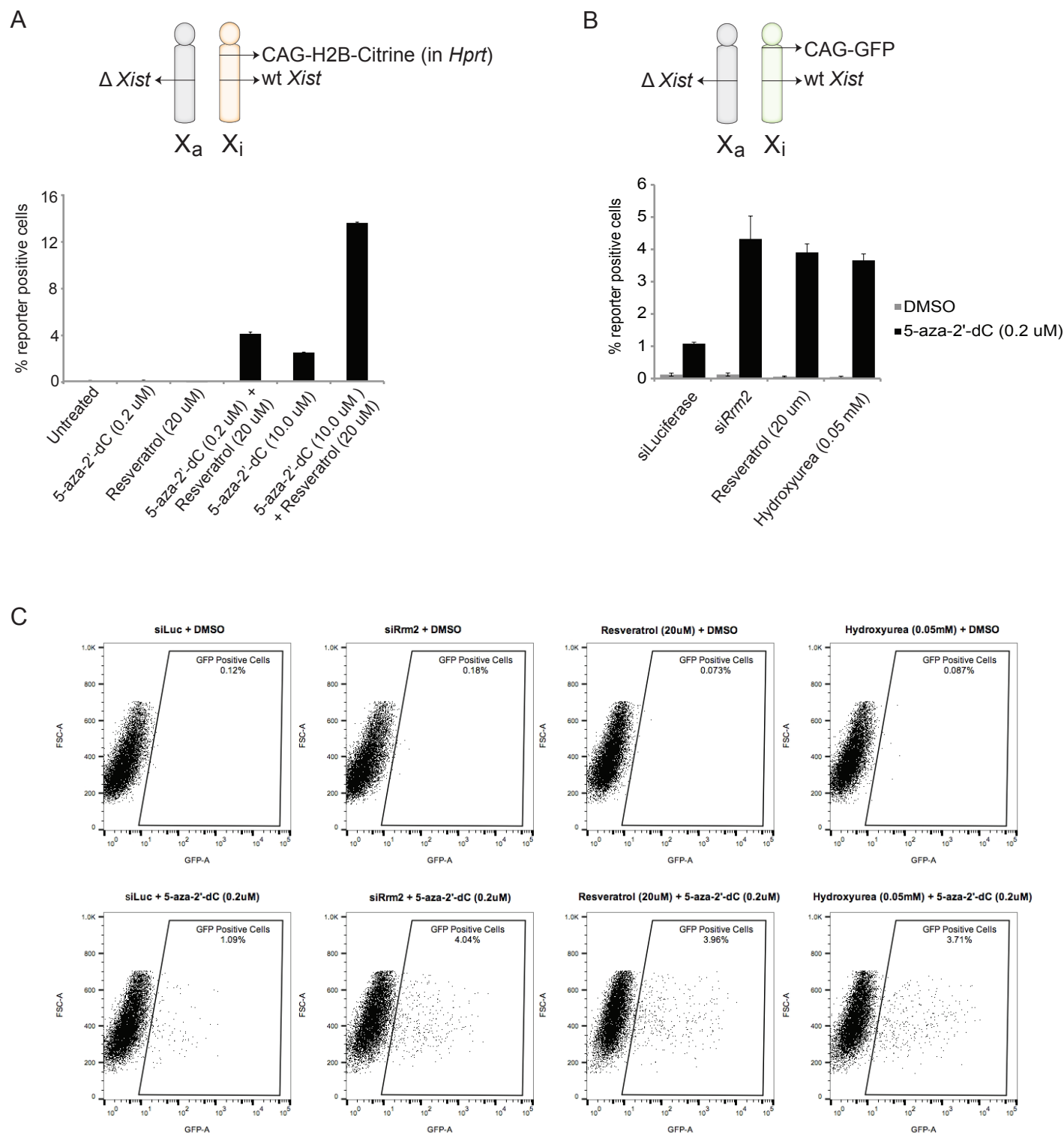

Supplement: Supplementary file 5 — 10.1186/s13072-015-0034-4 Validation of the resveratrol result with different Xi-reporter lines. A. Diagram of MEF Xi-H2B Citrine reporter genotype. As in Fig. 1A, except the Xi is bearing a CAG-driven histone H2B-Citrine reporter gene instead of luciferase in the Hprt locus The chart summarizes flow cytometry analysis of Xi-H2B Citrine reporter MEFs treated with resveratrol (20 μM) and/or 5-aza-2’-dC (0.2 μM or 10 μM) for 72 hours. B. Diagram of MEF Xi-GFP reporter genotype. The Xi is bearing a randomly integrated CAG-driven GFP allele near the centromere [58]. The chart summarizes flow cytometry analysis of GFP reporter MEFs treated with siRrm2, resveratrol (20 uM) or HU (0.05 mM), and DMSO or 5-aza-2’-dC (0.2 μM). Error bars represent standard deviation from triplicate wells. C. Representative flow cytometry dot plots of GFP reporter MEFs from part B. [file 13072_2015_34_MOESM5_ESM.pdf]

A

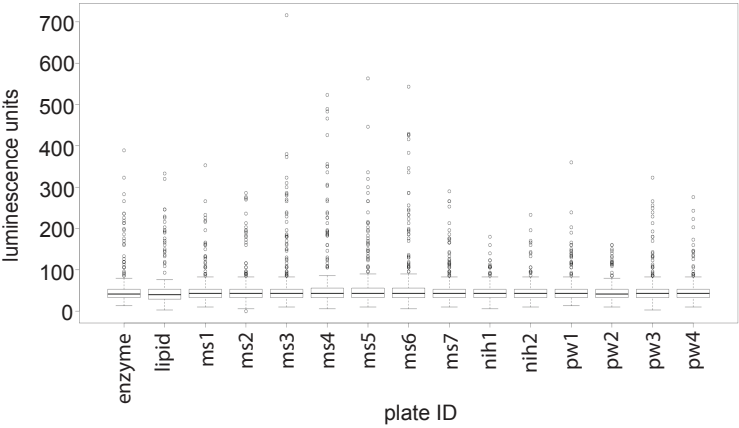

B

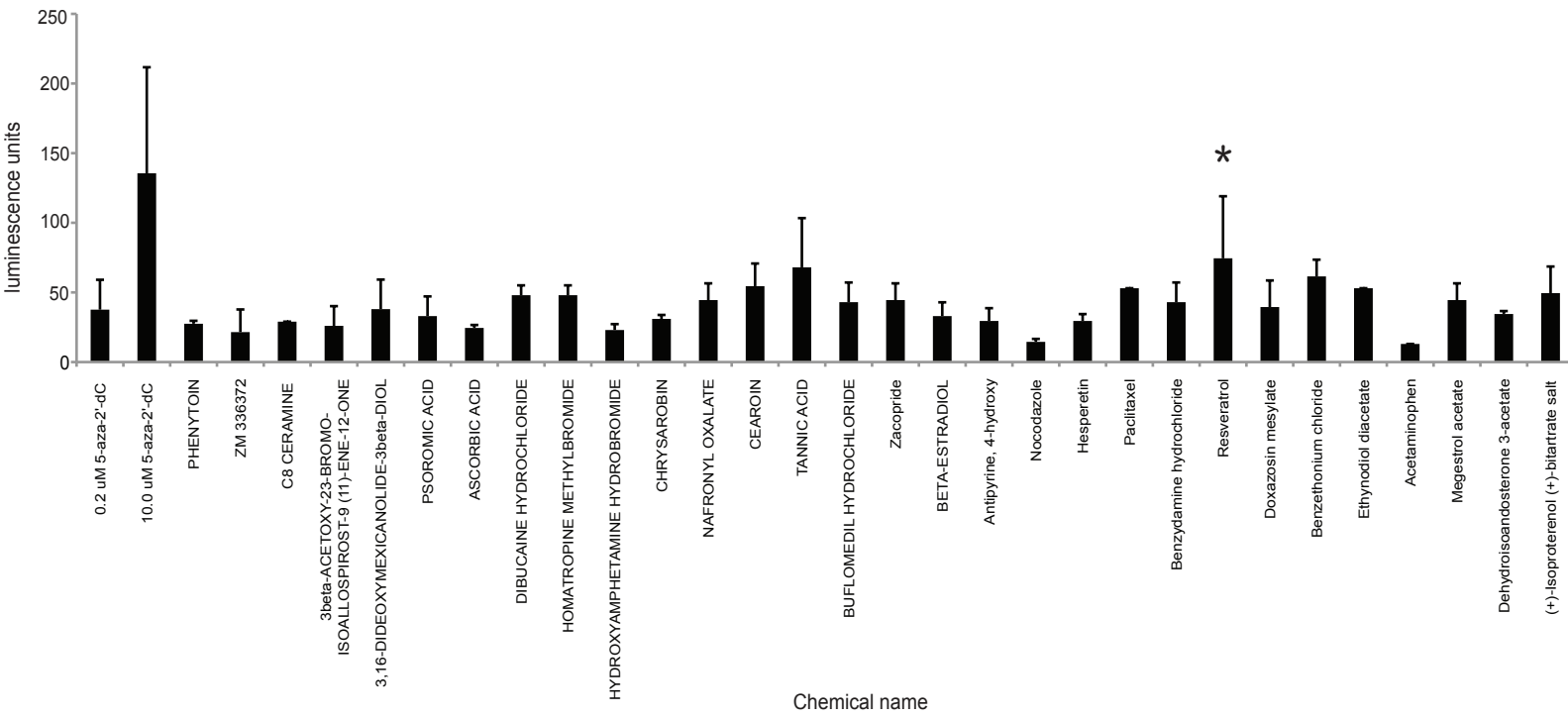

Supplement: Supplementary file 6 — 10.1186/s13072-015-0034-4 Chemical screen results and validation. A. Box plot of all raw luciferase measurements from the chemical screen by individual 384-well plate, demonstrating lack of obvious batch effect. Chemical library plates were prepared and assayed as one batch of 15 plates. B. Chart displaying results from the Xi-luciferase assay in the 24-well format upon treatment with various chemicals (at 10 μM) in the presence of 5-aza-2’-dC (0.2 μM) for 72 hours. Error bars indicate one standard deviation from duplicate wells except for negative control 5-aza-2’-dC (0.2 μM) alone (n=16) and positive control 5-aza-2’-dC (10.0 μM) alone (n=16). Resveratrol is indicated with an asterisk. [file 13072_2015_34_MOESM6_ESM.pdf]

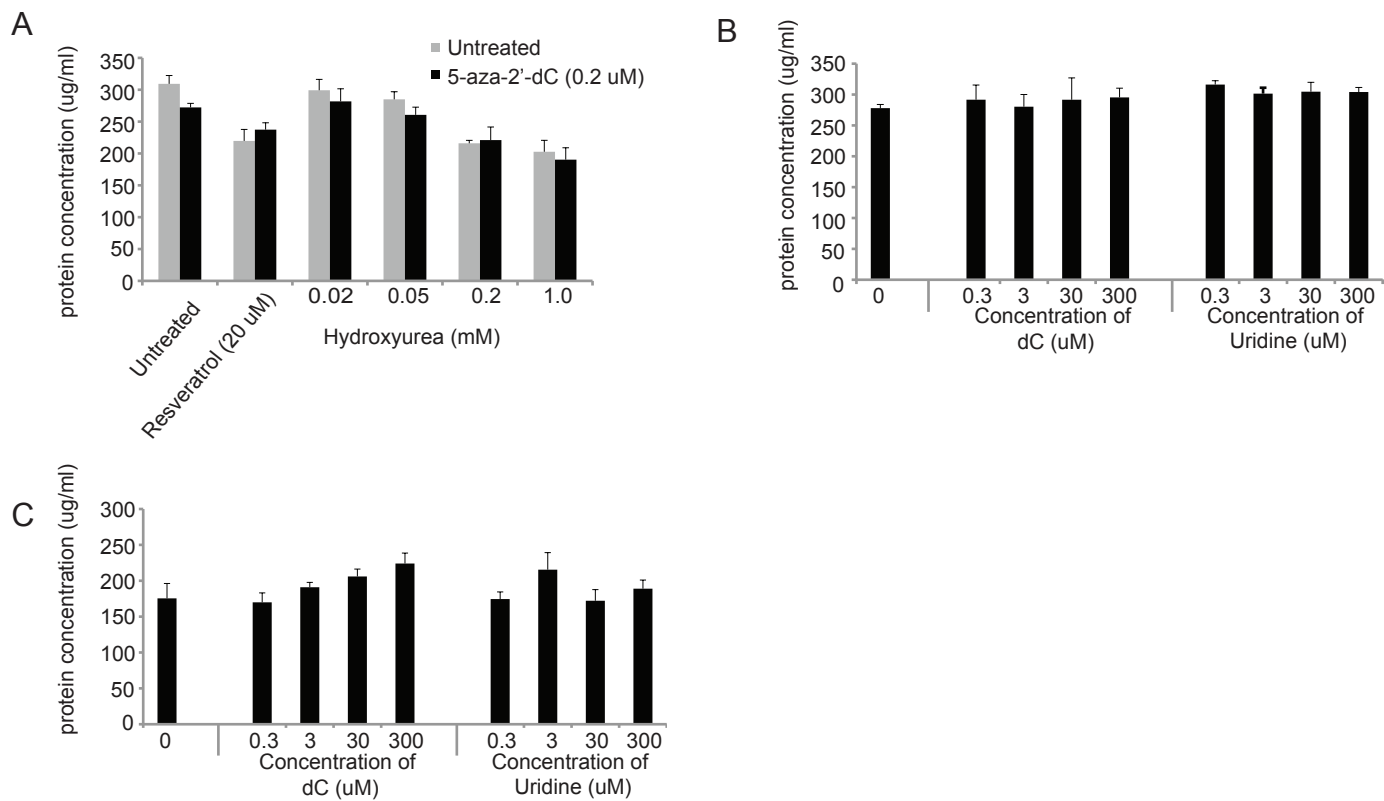

Supplement: Supplementary file 7 — 10.1186/s13072-015-0034-4 Protein concentration measurements for Xi-luciferase reactivation assays. A. Chart depicts protein concentration of cell lysates corresponding to luciferase measurements in (1G). Error bars indicate standard deviation from three individual wells. B. As in (A) but protein concentrations of cell lysates corresponding to luciferase measurements for (2C). C. As in (A) but protein concentrations of cell lysates corresponding to luciferase measurements for (2D). [file 13072_2015_34_MOESM7_ESM.pdf]

A

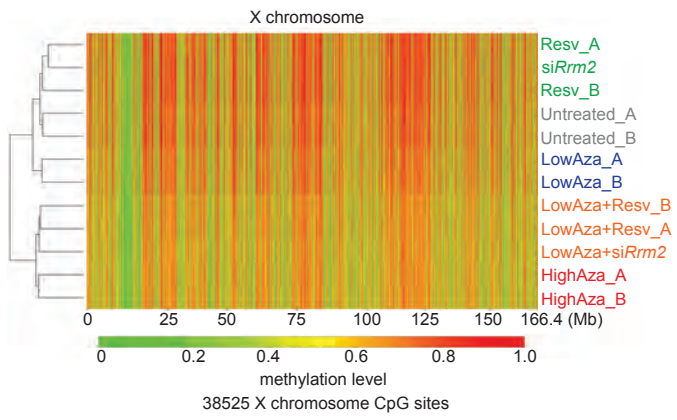

B i.

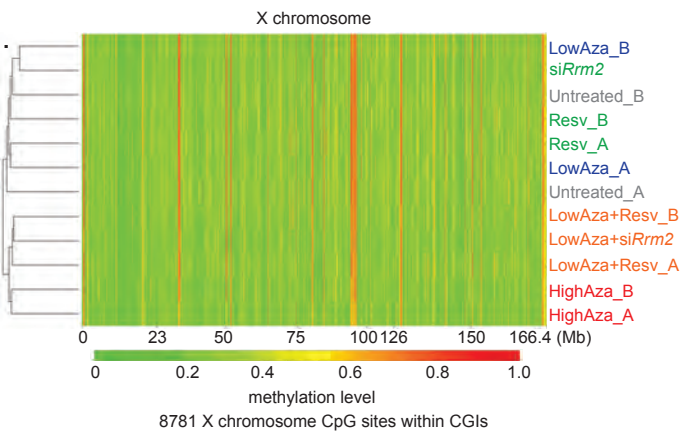

ii.

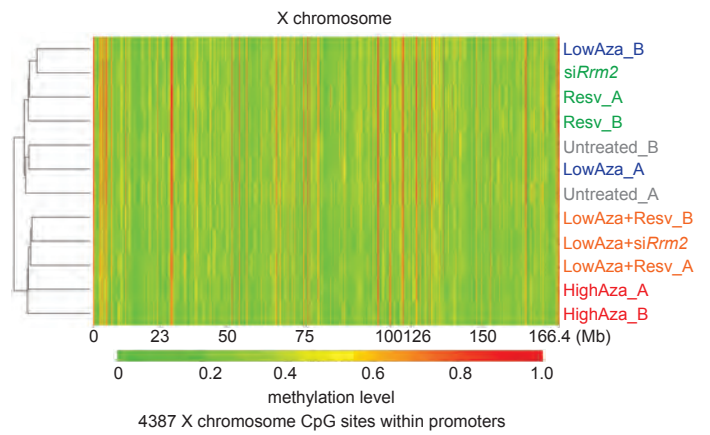

Supplement: Supplementary file 9 — 10.1186/s13072-015-0034-4 Analysis of DNA methylation status on the X chromosome in MEFs treated with combinations of RNR inhibition and 5-aza-2’-dC. A. Heat map of the unsupervised hierarchical clustering of CpG methylation levels in MEFs as in Fig. 3B, except that the data for X chromosome CpG sites are shown. B. (i) Heat map of the unsupervised hierarchical clustering as in Fig. 3B but only for CpG sites within CpG islands on the X chromosome. Constitutively hypermethylated (>0.75) and hypomethylated (<0.15) sites were filtered out to improve contrast. (ii) As in (i) except for CpG sites within promoters on the X chromosome. Again, constitutively hypermethylated (>0.75) and hypomethylated (<0.15) sites were filtered out to improve contrast. Promoters were defined as the region 1 kb upstream of the TSS for all UCSC genes. [file 13072_2015_34_MOESM9_ESM.pdf]

A

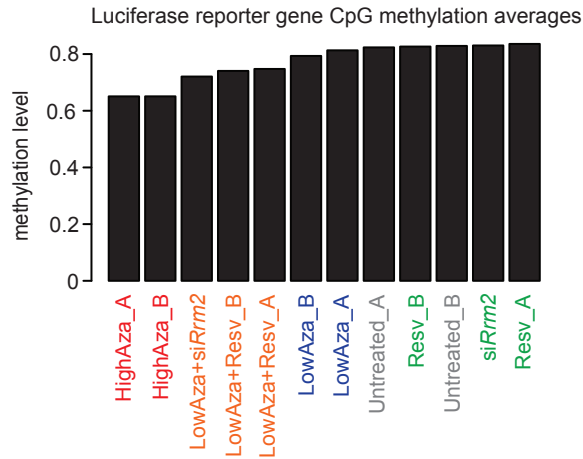

B

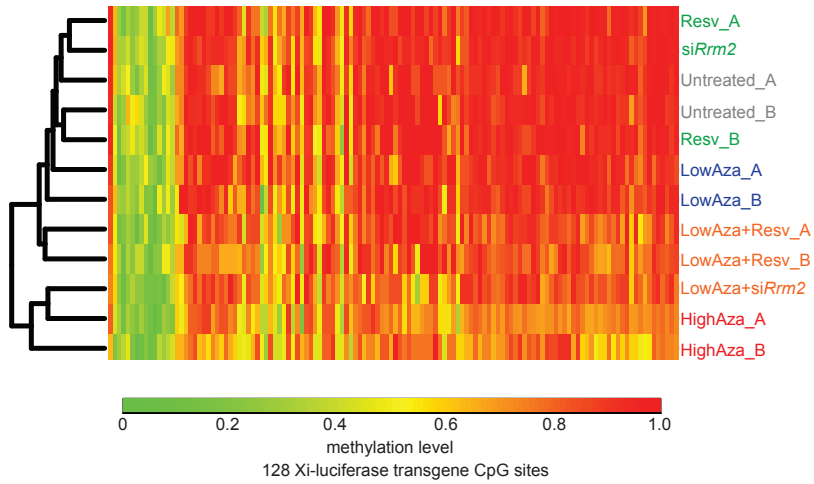

C

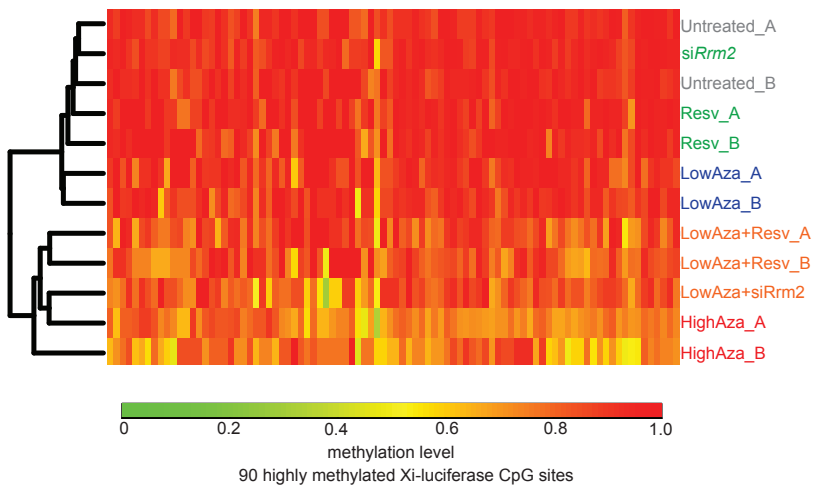

D

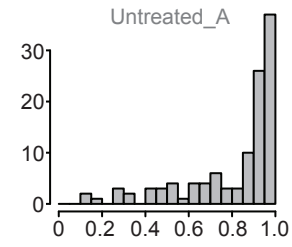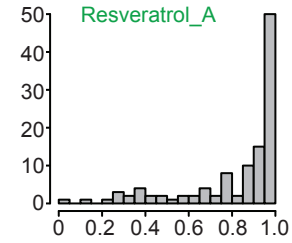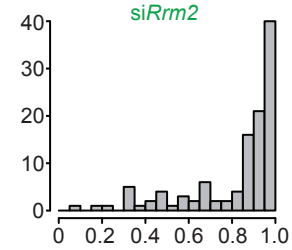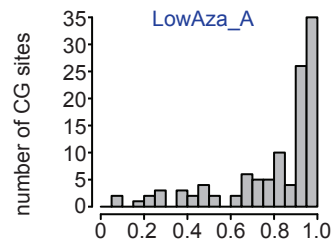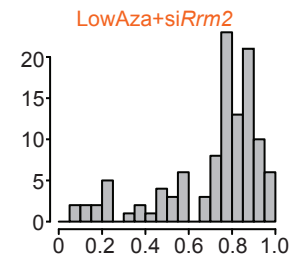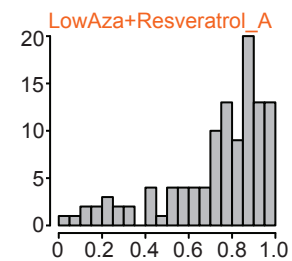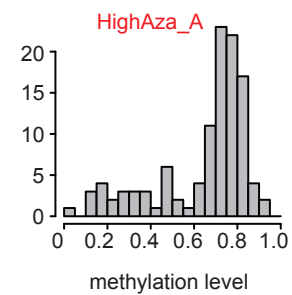

Supplement: Supplementary file 10 — 10.1186/s13072-015-0034-4 DNA methylation status of the luciferase transgene in MEFs treated with combinations of RNR inhibition and 5-aza-2’-dC. A. Bar chart displaying average CpG methylation levels for the indicated treatments filtered by CpGs with at least 5X sequencing coverage by RRBS across all samples as in Fig. 3A, but only considering the CpGs in the luciferase reporter gene/promoter. B. Heat map of unsupervised hierarchical clustering of CpG methylation levels as in Fig. 3B except for CpG sites in the luciferase reporter gene/promoter. C. As in (B), except for CpG sites within the luciferase reporter with a methylation level greater than 0.75 in both of the untreated samples. D. Histograms showing the distribution of CpG methylation levels within the luciferase reporter gene. [file 13072_2015_34_MOESM10_ESM.pdf]

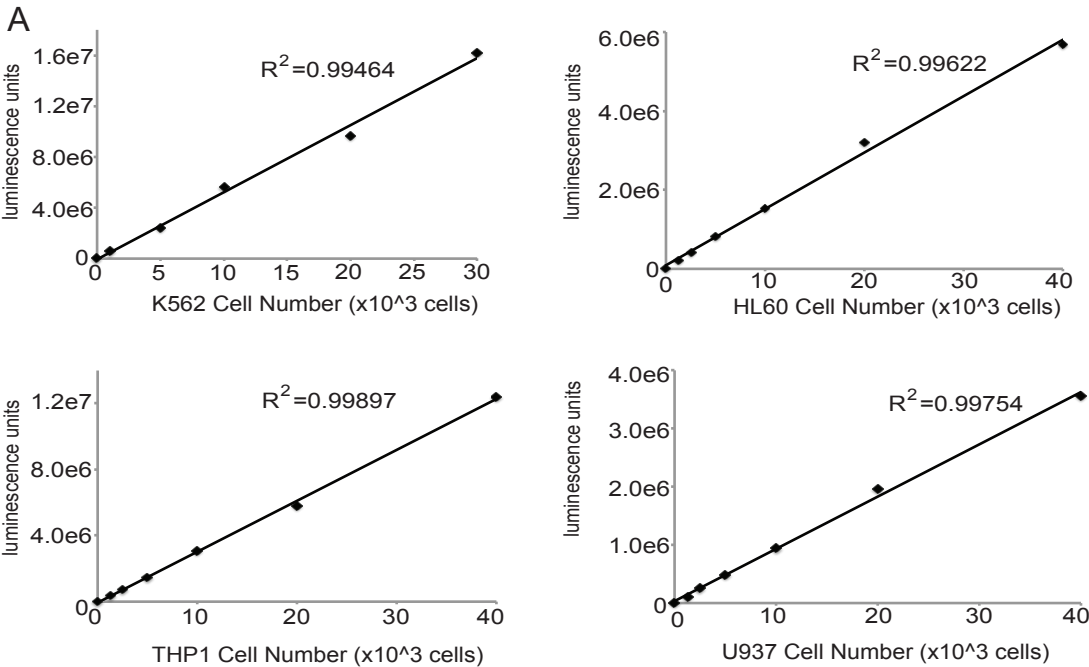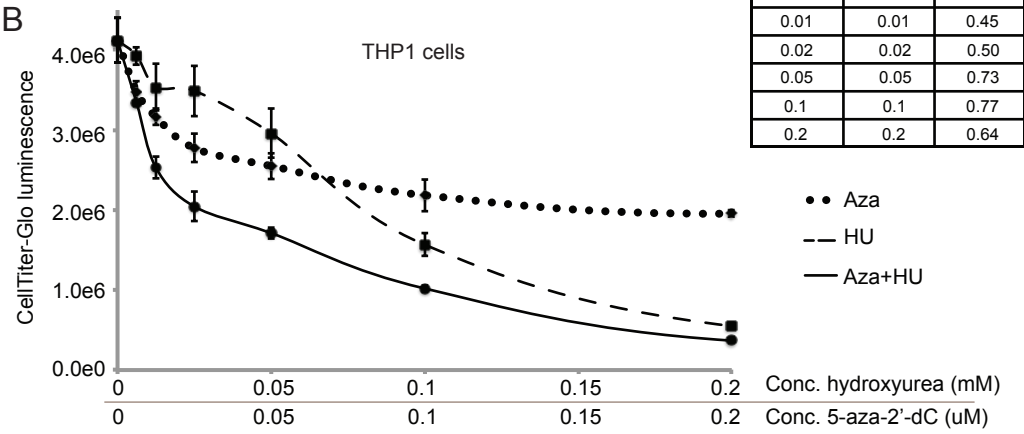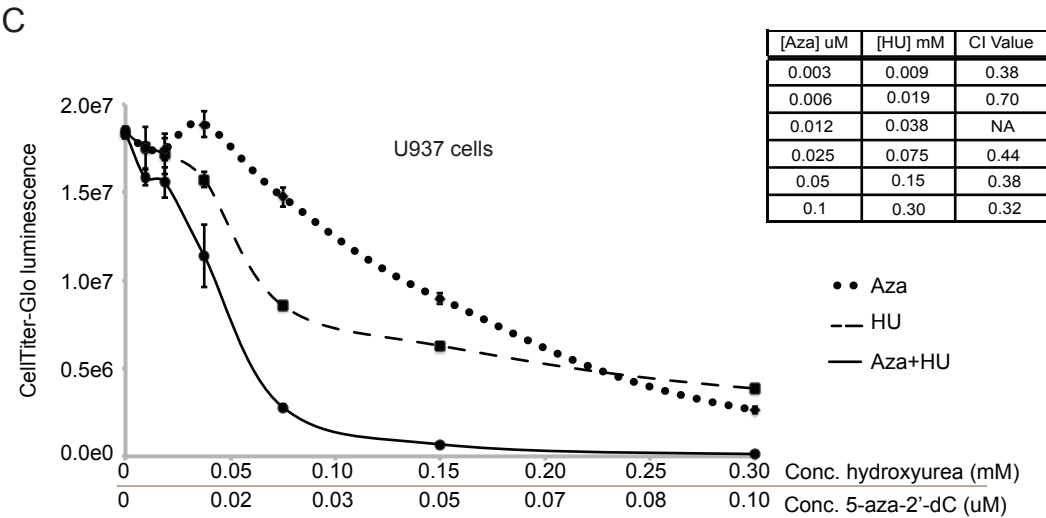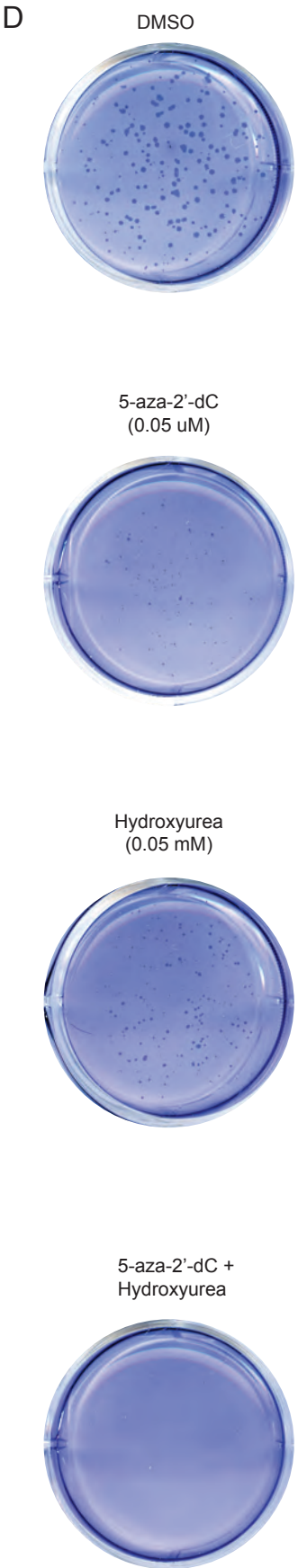

Supplement: Supplementary file 11 — 10.1186/s13072-015-0034-4 Synergistic effect of HU and 5-aza-2’dC on myeloid leukemia cell line proliferation. A. Graphs represent cell counts measured with the hemocytometer after trypan blue staining compared to viable cell number measurement determined by CellTiter-Glo reagent (Promega) for four myeloid leukemia cell lines. High correlation coefficient, R2, demonstrates linear relationship. B. Dose response curves as in Fig. 4A, except for THP1 cells using a fixed concentration ratio of 1000:1 HU:Aza. C. Dose response curves as in Fig. 4A, except for U937 cells using a fixed concentration ratio of 300:1 HU:Aza. D. Soft agar assay of K562 cells plated in DMSO or 5-aza-2’dC (0.05 uM) and/or HU (0.05 mM) in a final concentration of 3% agar and stained with crystal violet after 8 days of growth. [file 13072_2015_34_MOESM11_ESM.pdf]

A.

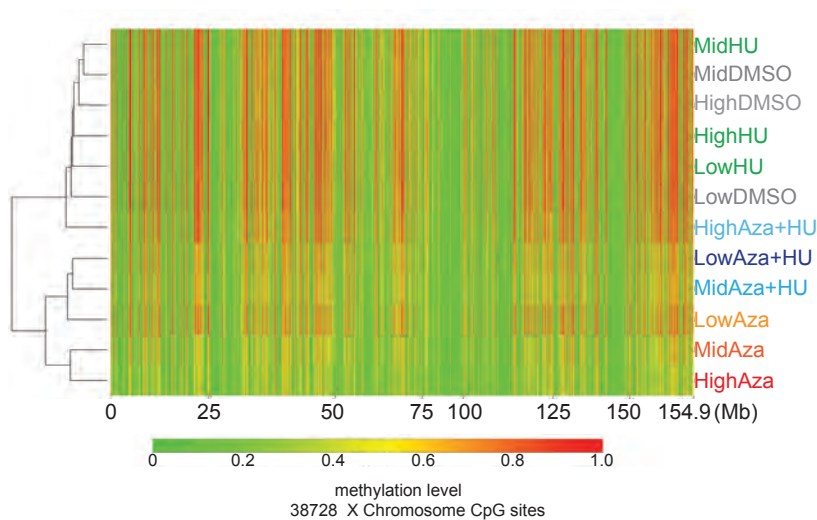

B.

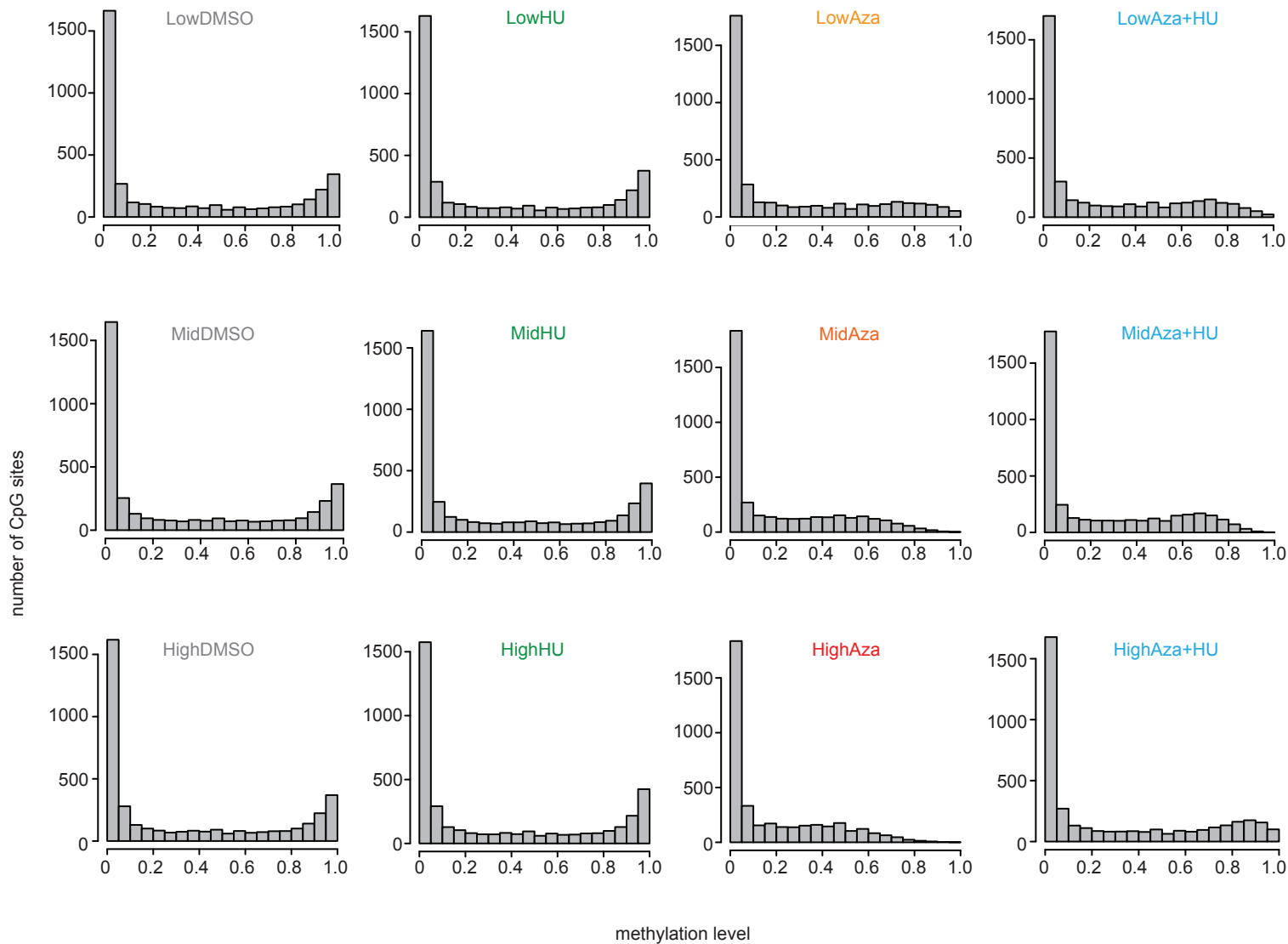

Supplement: Supplementary file 12 — 10.1186/s13072-015-0034-4 Extended data on the methylation analysis of K562 cells. A. Heat map showing an unsupervised hierarchical clustering of X chromosome CpG methylation in K562 cells treated with the indicated chemicals for 72 hours as in Fig. 4C but for all X chromosome CpGs. B. CpG methylation distribution along the X chromosome in K562 cells, for CpG sites with at least 10X coverage across all samples as determined by RRBS. Chemical treatments are as shown in Fig. 4A/C. [file 13072_2015_34_MOESM12_ESM.pdf]
