# Supplementary material for: A high-throughput screen of inactive X chromosome reactivation identifies the enhancement of DNA demethylation by 5-aza-2′-dC upon inhibition of ribonucleotide reductase
Source: Epigenetics Chromatin. 2015 Oct 13;8:42. doi: 10.1186/s13072-015-0034-4 (PMC4604769; doi:10.1186/s13072-015-0034-4)
Supplement: Supplementary file 3 — 10.1186/s13072-015-0034-4 Redundant siRNA activity ranking of genome-wide siRNA screen data. Table is color-coded to reflect which hit genes were validated. [file 13072_2015_34_MOESM3_ESM.pdf]

Supplementary Table 1: RSA of siRNA screen

Key		
Key	Internal control	siRNA.ID A and B reflect duplicate screens
	siRNA reordered	Rank represents order of activity by robust z-score
	Further validated	

	Gene.Symbol	Gene.ID	siRNA.ID	Score	LogP	Rank
1	Dnmt1	13433	161526B	7.6217	-4.82	201
1	Dnmt1	13433	161527B	3.8446	-4.82	1145
1	Dnmt1	13433	161527A	3.7771	-4.82	1217
1	Dnmt1	13433	161528A	2.4956	-4.82	3286
2	Cdkl3	213084	93379A	22.638	-4.73	12
2	Cdkl3	213084	93379B	7.9901	-4.73	180
3	1200006O19Rik	67452	80066B	3.5748	-4.70	1555
3	1200006O19Rik	67452	80066A	2.9003	-4.70	2232
3	1200006O19Rik	67452	179677A	2.5942	-4.70	3061
3	1200006O19Rik	67452	179676B	2.4089	-4.70	3527
4	C030011O14Rik	215708	171290A	8.6646	-4.68	131
4	C030011O14Rik	215708	95937B	3.5748	-4.68	1525
4	C030011O14Rik	215708	171290B	3.3206	-4.68	1815
4	C030011O14Rik	215708	171291A	2.2933	-4.68	3567
5	1600002K03Rik	69770	287617A	2.9003	-4.64	2231
5	1600002K03Rik	69770	287617B	2.9003	-4.64	2346
5	1600002K03Rik	69770	287616A	2.7461	-4.64	2755
5	1600002K03Rik	69770	287616B	2.2644	-4.64	3659
6	1700029F12Rik	66479	78478A	4.7214	-4.62	693
6	1700029F12Rik	66479	78664B	4.1083	-4.62	983
6	1700029F12Rik	66479	78478B	3.9951	-4.62	1097
7	0610039N19Rik	67442	80062A	6.2261	-4.50	343
7	0610039N19Rik	67442	79871A	4.0469	-4.50	1008
7	0610039N19Rik	67442	80062B	3.0352	-4.50	2051
8	Atf7ip	54343	176667B	20.909	-4.40	17
8	Atf7ip	54343	176667A	10.74	-4.40	84
8	Atf7ip	54343	73727A	3.6319	-4.40	1302
9	Gpr18	110168	174115B	87.212	-4.23	1
10	Ttc7	225049	169303B	1.9813	-4.17	5212
10	Ttc7	225049	169301B	1.7122	-4.17	6651
10	Ttc7	225049	169302A	1.6188	-4.17	6897
10	Ttc7	225049	169302B	1.5565	-4.17	7374
10	Ttc7	225049	169303A	1.3008	-4.17	10664
11	2210415K24Rik	67298	79629B	5.5516	-4.05	487
11	2210415K24Rik	67298	164913B	3.5748	-4.05	1564
11	2210415K24Rik	67298	79809A	3.3725	-4.05	1709
12	Slc7a6	330836	100159B	9.9246	-4.03	96
12	Slc7a6	330836	100159A	4.6696	-4.03	736
12	Slc7a6	330836	168793B	2.698	-4.03	2939
13	B3galt3	26879	74892B	5.5516	-4.02	497
13	B3galt3	26879	74985B	3.6319	-4.02	1325
13	B3galt3	26879	74892A	3.3725	-4.02	1752
14	2410018L13Rik	69732	283308B	7.2171	-4.00	238
14	2410018L13Rik	69732	283307B	6.9902	-4.00	267
15	AA986860	212439	171460A	7.6269	-3.89	196
15	AA986860	212439	98810B	2.2933	-3.89	3600
15	AA986860	212439	171460B	2.0235	-3.89	4931

15	AA986860	212439	98810A	1.8211	-3.89	5664
16	Efcbp2	117148	86107B	2.4956	-3.85	3430
16	Efcbp2	117148	163460B	2.0754	-3.85	4590
16	Efcbp2	117148	86107A	2.0235	-3.85	4859
16	Efcbp2	117148	86014A	1.8211	-3.85	5788
17	Dfna5h	54722	184889B	13.334	-3.78	49
17	Dfna5h	54722	184888A	6.2261	-3.78	341
18	Olfr1462	258688	283030A	7.5384	-3.77	208
18	Olfr1462	258688	283030B	5.2769	-3.77	547
19	Rps19	20085	152272A	46.335	-3.75	3
20	Eif2s1	13665	159512B	2.698	-3.74	2992
20	Eif2s1	13665	159511A	2.0235	-3.74	4851
20	Eif2s1	13665	159512A	2.0235	-3.74	4893
20	Eif2s1	13665	159511B	1.8211	-3.74	6197
21	Lmyc1	16918	62798B	5.5516	-3.71	494
21	Lmyc1	16918	155159A	3.6319	-3.71	1293
21	Lmyc1	16918	62798A	2.9003	-3.71	2223
22	Panx3	208098	93960B	32.376	-3.63	4
23	1110056N09Rik	68815	181149B	3.5748	-3.55	1629
23	1110056N09Rik	68815	181148B	2.9003	-3.55	2374
23	1110056N09Rik	68815	181147A	2.7498	-3.55	2512
24	8430437G11Rik	101118	174448A	28.574	-3.53	5
25	Slc35d2	70484	223118A	8.4571	-3.53	146
25	Slc35d2	70484	223116A	3.4243	-3.53	1676
25	Slc35d2	70484	223117A	2.7498	-3.53	2559
26	Zhx2	387609	169264A	3.7771	-3.49	1225
26	Zhx2	387609	169263A	2.5942	-3.49	3062
26	Zhx2	387609	169264B	2.0235	-3.49	5177
26	Zhx2	387609	169262A	1.5565	-3.49	7191
27	Calb3	12309	160954A	5.7072	-3.45	427
27	Calb3	12309	160954B	5.5516	-3.45	499
28	Stk19	54402	73555A	25.159	-3.45	6
29	MGC69750	245670	254262B	5.1936	-3.44	569
29	MGC69750	245670	254263A	4.3064	-3.44	852
29	MGC69750	245670	254263B	2.7498	-3.44	2741
30	Treh	58866	181964B	7.2171	-3.41	252
30	Treh	58866	181964A	5.3959	-3.41	523
31	Ogt	108155	88401A	24.956	-3.39	7
32	Rev1l	56210	74011A	9.1538	-3.38	112
32	Rev1l	56210	74011B	5.3538	-3.38	543
33	Mrpl53	68499	81035A	4.6315	-3.38	754
33	Mrpl53	68499	180554A	4.0469	-3.38	1001
33	Mrpl53	68499	102282B	2.698	-3.38	2875
34	Olfr958	258327	103129A	5.5516	-3.38	475
34	Olfr958	258327	91059B	4.0469	-3.38	1043
34	Olfr958	258327	165082A	1.9197	-3.38	5303
34	Olfr958	258327	91059A	1.5513	-3.38	7671
35	Brunol4	108013	172618B	2.8819	-3.38	2428
35	Brunol4	108013	172618A	2.2764	-3.38	3626
35	Brunol4	108013	172617A	1.8211	-3.38	5782
35	Brunol4	108013	172619A	1.5513	-3.38	7679
36	9030603L14Rik	67564	80316A	9.6473	-3.33	106
36	9030603L14Rik	67564	80316B	5.1884	-3.33	573
37	Hectd2	226098	94601A	23.812	-3.33	8
38	43J09Rik	73167	83091A	3.3725	-3.31	1720

38	3110043J09Rik	73167	82904B	2.434	-3.31	3489
38	3110043J09Rik	73167	83091B	2.0235	-3.31	5052
38	3110043J09Rik	73167	177697B	1.5513	-3.31	8008
39	1700012B15Rik	74173	83205B	7.4194	-3.30	213
39	1700012B15Rik	74173	177954B	2.698	-3.30	2920
40	12B15Rik	74173	83397A	2.5942	-3.30	3056
41	Axin2	12006	161735A	23.348	-3.28	9
42	Magee1	107528	85931B	3.3725	-3.26	1764
42	Magee1	107528	85757B	3.1797	-3.26	1897
42	Magee1	107528	172234A	2.4956	-3.26	3160
43	Wbscr18	66114	78044A	3.5748	-3.25	1428
43	Wbscr18	66114	78128B	1.9197	-3.25	5427
43	Wbscr18	66114	78128A	1.5565	-3.25	7220
43	Wbscr18	66114	182701B	1.5513	-3.25	8299
44	C030022K24Rik	77462	84195A	6.5893	-3.24	294
44	C030022K24Rik	77462	84195B	4.8771	-3.24	662
44	C030022K24Rik	77462	174674A	2.4956	-3.24	3208
45	Cdca8	52276	261273B	7.7826	-3.24	189
45	Cdca8	52276	80744B	3.5748	-3.24	1531
45	Cdca8	52276	80839A	2.4956	-3.24	3211
46	Echs1	93747	85503B	23.337	-3.23	10
47	2410129H14Rik	76789	176221B	3.3725	-3.23	1776
47	2410129H14Rik	76789	176221A	1.7641	-3.23	6356
47	2410129H14Rik	76789	176220A	1.5513	-3.23	7488
47	2410129H14Rik	76789	176220B	1.4716	-3.23	8371
48	Dhcr24	74754	85973A	5.3959	-3.22	520
48	Dhcr24	74754	85973B	2.698	-3.22	2953
48	Dhcr24	74754	86069A	2.4956	-3.22	3264
49	Syngr4	58867	75846A	6.2728	-3.20	329
49	Syngr4	58867	75939A	4.8252	-3.20	672
50	Atpaf2	246782	89344A	2.5942	-3.13	3021
50	Atpaf2	246782	89344B	1.8211	-3.13	5939
50	Atpaf2	246782	152670A	1.4009	-3.13	8589
50	Atpaf2	246782	152670B	1.4009	-3.13	8895
51	Caskin2	140721	86361B	20.235	-3.13	19
52	B430306N03Rik	320148	154353B	22.258	-3.12	13
53	4933409N07Rik	71069	178823A	4.8771	-3.11	659
53	4933409N07Rik	71069	96186B	2.9003	-3.11	2326
53	4933409N07Rik	71069	96186A	2.3461	-3.11	3547
54	Enpp2	18606	72080B	4.2493	-3.11	925
54	Enpp2	18606	72176A	2.0235	-3.11	4860
54	Enpp2	18606	72270A	1.8211	-3.11	5884
54	Enpp2	18606	72270B	1.4009	-3.11	9020
55	1200009K13Rik	66870	79219A	22.216	-3.09	14
56	Olfr676	259099	92240A	2.2258	-3.09	3882
56	Olfr676	259099	92379B	2.0235	-3.09	4952
56	Olfr676	259099	92324B	2.0235	-3.09	5091
56	Olfr676	259099	92379A	1.349	-3.09	9134
57	Kcnk2	16526	155982B	5.6368	-3.06	443
57	Kcnk2	16526	67649B	3.7771	-3.06	1247
58	Bin3	57784	75529B	4.8252	-3.05	680
58	Bin3	57784	75433A	4.4516	-3.05	794
59	Adcy5	224129	259754B	4.9238	-3.05	643
59	Adcy5	224129	259753B	2.4956	-3.05	3422
59	Adcy5	224129	259753A	2.231	-3.05	3731

60	Creb5	231991	94872A	6.2728	-3.05	327
60	Creb5	231991	94872B	4.4516	-3.05	800
61	Ms4a4d	66607	78717B	5.5983	-3.04	461
61	Ms4a4d	66607	78717A	3.6899	-3.04	1268
62	Try10l	386551	254201A	2.1078	-3.03	4220
62	Try10l	386551	286929B	2.0235	-3.03	5043
62	Try10l	386551	254201B	1.7641	-3.03	6381
63	Zfp97	22759	287481A	5.5983	-3.03	448
63	Zfp97	22759	69935A	2.7498	-3.03	2464
63	Zfp97	22759	287481B	2.231	-3.03	3780
64	31G14Rik	66736	183337B	21.786	-3.03	16
65	Osbpl10	74486	178687B	3.113	-3.02	2007
65	Osbpl10	74486	92433A	2.9055	-3.02	2126
65	Osbpl10	74486	92433B	2.231	-3.02	3806
66	Steap	70358	81975B	1.9197	-3.02	5378
66	Steap	70358	81785B	1.7537	-3.02	6460
66	Steap	70358	180981A	1.5513	-3.02	7520
66	Steap	70358	81785A	1.349	-3.02	9515
67	2900009I07Rik	68034	102183A	2.9574	-3.01	2084
67	2900009I07Rik	68034	180292B	2.4956	-3.01	3318
67	2900009I07Rik	68034	180291A	2.2258	-3.01	3839
68	Rps6ka4	56613	74802A	4.5107	-3.01	780
68	Rps6ka4	56613	74802B	3.6319	-3.01	1318
69	Trim8	93679	175426B	2.9055	-2.99	2162
69	Trim8	93679	175427B	2.0754	-2.99	4597
69	Trim8	93679	175427A	1.5513	-2.99	7895
69	Trim8	93679	85675A	1.349	-2.99	9652
70	Il2rb	16185	158301A	4.4516	-2.99	799
70	Il2rb	16185	158300B	3.2687	-2.99	1873
70	Il2rb	16185	158300A	1.8211	-2.99	5862
70	Il2rb	16185	62402A	1.349	-2.99	9671
71	Sf3b2	319322	152516A	19.26	-2.99	21
72	Tktl1	83553	175120B	2.2258	-2.99	4200
72	Tktl1	83553	175120A	2.0235	-2.99	4927
72	Tktl1	83553	85091B	1.7122	-2.99	6714
72	Tktl1	83553	84910A	1.349	-2.99	9684
73	Ankrd32	105377	172827A	20.707	-2.98	18
74	4631402N15Rik	70793	167532B	7.4194	-2.98	214
74	4631402N15Rik	70793	96016A	4.2493	-2.98	869
75	9530080O11Rik	319247	97186A	7.1081	-2.97	257
75	9530080O11Rik	319247	153971A	4.2493	-2.97	877
76	2610020C11Rik	72154	82719A	2.9003	-2.96	2241
76	2610020C11Rik	72154	82719B	2.7498	-2.96	2669
76	2610020C11Rik	72154	177451A	2.2258	-2.96	4008
77	Stac3	237611	98893B	1.8211	-2.95	6106
77	Stac3	237611	168325B	1.7122	-2.95	6619
77	Stac3	237611	99085A	1.5565	-2.95	7034
77	Stac3	237611	99085B	1.349	-2.95	9888
78	MOR135-11	258925	283107A	2.8329	-2.95	2439
78	MOR135-11	258925	283108A	2.2258	-2.95	3881
78	MOR135-11	258925	288091A	2.0235	-2.95	4641
78	MOR135-11	258925	288091B	1.349	-2.95	9926
79	Mrpl49	18120	80321A	2.8329	-2.95	2440
79	Mrpl49	18120	157139A	2.0235	-2.95	4642
79	Mrpl49	18120	80321B	1.9271	-2.95	5253

79	Mrpl49	18120	157139B	1.349	-2.95	9930
80	Nanos1	332397	99631A	4.7964	-2.94	685
80	Nanos1	332397	99721B	3.1797	-2.94	1907
80	Nanos1	332397	168744B	2.0754	-2.94	4521
80	Nanos1	332397	99631B	1.349	-2.94	9937
81	3110052N05Rik	76987	174627B	3.0352	-2.94	2056
81	3110052N05Rik	76987	174627A	2.5942	-2.94	3027
81	3110052N05Rik	76987	84076A	1.6188	-2.94	6886
82	Bcdo2	170752	172667B	3.1797	-2.90	1894
82	Bcdo2	170752	86555A	2.698	-2.90	2817
82	Bcdo2	170752	172667A	1.5565	-2.90	7059
83	4632423N09Rik	103861	87611A	8.566	-2.90	132
83	4632423N09Rik	103861	87611B	4.1936	-2.90	948
84	2610003J06Rik	72106	177421A	3.113	-2.90	1953
84	2610003J06Rik	72106	82606B	1.4716	-2.90	8373
84	2610003J06Rik	72106	177420A	1.4009	-2.90	8510
84	2610003J06Rik	72106	177421B	1.349	-2.90	10206
85	1110036O03Rik	66180	183998B	6.377	-2.89	320
85	1110036O03Rik	66180	183999A	2.2258	-2.89	3918
85	1110036O03Rik	66180	183998A	2.1078	-2.89	4221
86	45E13Rik	208924	166525B	18.683	-2.89	22
87	B430104H02Rik	68126	100874A	3.5748	-2.88	1419
87	B430104H02Rik	68126	100952A	3.0834	-2.88	2029
87	B430104H02Rik	68126	100874B	2.0821	-2.88	4257
88	AI316787	195040	257576B	4.9238	-2.88	632
88	AI316787	195040	174178A	2.2933	-2.88	3563
88	AI316787	195040	257576A	2.0754	-2.88	4263
89	Slamf6	30925	84634A	7.2171	-2.88	233
89	Slamf6	30925	186252B	4.1507	-2.88	975
90	C030018G13Rik	241076	153800A	2.2933	-2.88	3568
90	C030018G13Rik	241076	153799A	2.231	-2.88	3710
90	C030018G13Rik	241076	97118A	2.0754	-2.88	4282
91	4930470P17Rik	67637	80256A	18.414	-2.87	23
92	Asf1a	66403	78442B	18.009	-2.85	24
93	4930518C23Rik	319210	97089B	15.041	-2.84	37
94	Apxl	110380	173535A	2.0235	-2.84	4858
94	Apxl	110380	173536A	1.4009	-2.84	8720
94	Apxl	110380	93948B	1.349	-2.84	10409
94	Apxl	110380	173536B	1.349	-2.84	10589
95	A230083G16Rik	442825	219935A	4.8771	-2.83	653
95	A230083G16Rik	442825	219935B	4.0469	-2.83	1026
96	4632412N22Rik	320159	167602A	9.1731	-2.83	111
96	4632412N22Rik	320159	97559B	4.0469	-2.83	1031
97	Prkcb1	18751	150143B	6.1877	-2.83	356
97	Prkcb1	18751	150142A	2.2258	-2.83	3914
97	Prkcb1	18751	63728B	1.9008	-2.83	5477
97	Prkcb1	18751	63728A	1.3008	-2.83	10667
98	Vps29	56433	74440A	4.0469	-2.82	1012
98	Vps29	56433	74345B	4.0469	-2.82	1039
99	2210408I21Rik	72371	166108A	2.9003	-2.82	2205
99	2210408I21Rik	72371	166108B	1.9197	-2.82	5365
99	2210408I21Rik	72371	166107A	1.4009	-2.82	8572
99	2210408I21Rik	72371	166106B	1.2877	-2.82	10705
100	H2-Ke6	14979	159272A	17.485	-2.82	26
101	Mafg	17134	101951B	3.7875	-2.80	1179

101	Mafg	17134	100342A	2.7498	-2.80	2463
101	Mafg	17134	156223B	2.0754	-2.80	4539
102	Myst2	217127	171478B	17.132	-2.80	27
103	9430007A20Rik	381572	164503B	4.3535	-2.80	836
103	9430007A20Rik	381572	164505B	4.0176	-2.80	1066
104	Olfr6	233670	166517A	2.698	-2.79	2796
104	Olfr6	233670	257627A	1.7122	-2.79	6497
104	Olfr6	233670	166518B	1.6188	-2.79	6946
104	Olfr6	233670	166518A	1.2452	-2.79	10909
105	Ube4b	63958	76450B	3.5748	-2.79	1588
105	Ube4b	63958	182218B	3.5748	-2.79	1601
105	Ube4b	63958	182219B	2.0754	-2.79	4591
106	Crry	12946	161688B	3.5748	-2.79	1583
106	Crry	12946	70968B	2.9003	-2.79	2317
106	Crry	12946	161688A	1.5513	-2.79	7736
107	Myod1	17927	68402A	3.5748	-2.79	1464
107	Myod1	17927	68309B	2.9003	-2.79	2368
107	Myod1	17927	156429B	2.0754	-2.79	4592
108	Slc9a3r2	65962	76743B	17.122	-2.78	28
109	Olfr202	258997	92157A	4.0469	-2.78	1010
109	Olfr202	258997	263130B	3.9951	-2.78	1098
110	Arid5a	214855	90597B	2.9003	-2.77	2311
110	Arid5a	214855	90502A	1.8211	-2.77	5564
110	Arid5a	214855	90597A	1.2452	-2.77	10810
110	Arid5a	214855	163701B	1.2452	-2.77	11024
111	Dusp6	67603	80339B	17.065	-2.77	29
112	15B01Rik	58909	183939A	16.66	-2.75	30
113	Zfy1	22767	262850A	3.8446	-2.75	1133
113	Zfy1	22767	65439A	3.113	-2.75	1941
113	Zfy1	22767	262849A	2.0235	-2.75	4718
114	Cnnm1	83674	175155B	4.0176	-2.75	1064
114	Cnnm1	83674	175155A	3.8446	-2.75	1128
115	C630013N10Rik	234378	93518B	3.5748	-2.74	1530
115	C630013N10Rik	234378	93710B	2.7498	-2.74	2597
115	C630013N10Rik	234378	93614A	2.0235	-2.74	4760
116	Semcap2	54120	162580A	16.188	-2.74	31
117	Olfr1271	258789	102432A	3.5748	-2.74	1364
117	Olfr1271	258789	91710B	2.4282	-2.74	3505
117	Olfr1271	258789	91710A	2.0235	-2.74	4772
118	Dcl1	93675	75086B	13.49	-2.74	47
119	Wdr8	59002	181987A	4.2493	-2.73	873
119	Wdr8	59002	75764A	2.7498	-2.73	2489
119	Wdr8	59002	75951A	2.0235	-2.73	4796
120	Ankrd6	140577	172460B	15.716	-2.73	32
121	Sc4mol	66234	182825A	5.5516	-2.72	474
121	Sc4mol	66234	182825B	3.7875	-2.72	1172
122	Ripk3	56532	185797B	3.7875	-2.72	1186
122	Ripk3	56532	185797A	3.5748	-2.72	1477
122	Ripk3	56532	185795A	2.0235	-2.72	4861
123	2810453L12Rik	72776	262088A	15.565	-2.71	33
124	Il12rb2	16162	62484A	3.1085	-2.71	2012
124	Il12rb2	16162	62484B	2.7498	-2.71	2682
124	Il12rb2	16162	62576A	2.0235	-2.71	4874
125	Cp	12870	160325B	3.2687	-2.71	1874
125	Cp	12870	60889A	2.3461	-2.71	3549

125	Cp	12870	160325A	2.0235	-2.71	4877
126	Dhrs4	28200	84610B	5.8681	-2.71	410
126	Dhrs4	28200	186247B	3.7875	-2.71	1188
127	Nfx1	74164	176785B	15.565	-2.70	34
128	Olfr661	258743	91683B	5.6657	-2.69	441
128	Olfr661	258743	165296A	3.7771	-2.69	1209
129	Th	21823	186734A	5.8681	-2.68	398
129	Th	21823	186732B	3.1701	-2.68	1927
130	BC008103	229524	88892B	2.4956	-2.68	3393
130	BC008103	229524	163647A	2.0754	-2.68	4284
130	BC008103	229524	88987B	2.0235	-2.68	4993
131	Inhba	16323	62600B	4.6696	-2.68	750
131	Inhba	16323	158325A	3.7771	-2.68	1227
132	Reg1	19692	150526B	5.5516	-2.68	498
132	Reg1	19692	150527A	3.7771	-2.68	1228
133	BC021891	234878	152720B	3.6319	-2.68	1316
133	BC021891	234878	89894A	1.7641	-2.68	6353
133	BC021891	234878	152720A	1.4333	-2.68	8431
134	A930026L03Rik	243382	96940B	15.311	-2.68	36
135	Prss3	22073	69663B	12.815	-2.68	54
136	4933400F01Rik	232973	164328B	3.9951	-2.67	1091
136	4933400F01Rik	232973	164326B	3.7771	-2.67	1235
137	Scamp1	107767	258830A	6.3113	-2.67	326
137	Scamp1	107767	258832B	2.0821	-2.67	4254
137	Scamp1	107767	258831B	2.0235	-2.67	5029
138	Rspondin	192199	88361A	2.4956	-2.67	3173
138	Rspondin	192199	88171B	2.231	-2.67	3768
138	Rspondin	192199	88267A	1.7986	-2.67	6327
139	Cyp11a1	13070	74531B	11.197	-2.66	70
139	Cyp11a1	13070	161851B	3.7771	-2.66	1254
140	Insl5	23919	70175A	2.5942	-2.66	3029
140	Insl5	23919	70363A	2.0235	-2.66	4691
140	Insl5	23919	70270B	2.0235	-2.66	5087
141	Olfr178	258999	102646A	2.4282	-2.66	3500
141	Olfr178	258999	102552A	2.0235	-2.66	4699
141	Olfr178	258999	102646B	2.0235	-2.66	5097
142	Tcfcp2l1	81879	77330B	14.839	-2.65	38
143	Rad51c	114714	172295A	3.8446	-2.65	1136
143	Rad51c	114714	86067B	3.679	-2.65	1274
144	Slitrk6	239250	153779A	14.367	-2.64	39
145	Rce1	19671	76766B	7.4713	-2.64	209
145	Rce1	19671	76766A	3.0774	-2.64	2032
146	1700129L13Rik	67933	180212B	2.8819	-2.63	2427
146	1700129L13Rik	67933	180211A	2.4956	-2.63	3242
146	1700129L13Rik	67933	180212A	1.9813	-2.63	5204
147	2810468K05Rik	216965	88822A	14.367	-2.63	40
148	F7	14068	158466A	2.9509	-2.63	2107
148	F7	14068	158464B	2.2258	-2.63	4126
148	F7	14068	158466B	1.4009	-2.63	8756
149	Tbx21	57765	181596A	5.8629	-2.63	415
149	Tbx21	57765	73888A	3.6319	-2.63	1304
150	9130014G24	215772	94333A	5.1027	-2.62	580
150	9130014G24	215772	167306B	3.6319	-2.62	1319
151	Olfr699	258180	283642B	10.792	-2.62	81
151	Olfr699	258180	283641B	3.6319	-2.62	1320

152	Olfr73	117004	86195A	3.6319	-2.62	1298
152	Olfr73	117004	86195B	3.6319	-2.62	1321
153	V1rc21	171194	87708B	8.566	-2.61	144
153	V1rc21	171194	87796B	2.4956	-2.61	3353
153	V1rc21	171194	87796A	1.9197	-2.61	5278
154	2410018C17Rik	74504	179041B	2.8819	-2.61	2411
154	2410018C17Rik	74504	179040A	2.0754	-2.61	4335
154	2410018C17Rik	74504	179039A	1.9197	-2.61	5293
155	Ascc2	75452	83631A	13.853	-2.61	42
156	Opn1sw	12057	159834B	4.2493	-2.61	937
156	Opn1sw	12057	159835B	3.6319	-2.61	1335
157	BC011209	217721	169756B	4.6733	-2.61	733
157	BC011209	217721	169756A	2.9055	-2.61	2109
158	Olfr627	436002	287161B	13.853	-2.60	43
159	Olfr463	258408	283090B	5.5516	-2.59	489
159	Olfr463	258408	283090A	3.5748	-2.59	1362
160	Il1rl1l	17083	156189A	13.692	-2.59	44
161	A230098A12Rik	235472	153752B	5.5516	-2.59	492
161	A230098A12Rik	235472	153752A	3.5748	-2.59	1368
162	U2af2	22185	86997B	2.4956	-2.58	3365
162	U2af2	22185	86808A	1.9813	-2.58	5203
162	U2af2	22185	188973B	1.9197	-2.58	5399
163	4933425I22Rik	71148	258780B	2.231	-2.58	3824
163	4933425I22Rik	71148	258780A	2.2258	-2.58	4009
163	4933425I22Rik	71148	258778B	1.9197	-2.58	5405
164	Abcc5	27416	256541B	11.563	-2.58	67
165	Tmod3	50875	72934B	8.0939	-2.58	174
165	Tmod3	50875	184724A	3.5748	-2.58	1379
166	Hist1h1a	80838	84598B	4.5375	-2.57	757
166	Hist1h1a	80838	84693A	3.5748	-2.57	1391
167	2610304F09Rik	269003	153510B	13.551	-2.57	46
168	Ndufs4	17993	156465B	6.3817	-2.55	319
168	Ndufs4	17993	68230A	3.5748	-2.55	1421
169	Mare	17168	157247A	13.355	-2.55	48
170	Apg4a	103003	166322A	2.231	-2.55	3683
170	Apg4a	103003	166321B	2.0754	-2.55	4532
170	Apg4a	103003	166323A	1.8211	-2.55	5563
171	Esx1	13984	157396B	4.4516	-2.54	821
171	Esx1	13984	61425A	3.5748	-2.54	1438
172	LOC433332	433332	217827B	3.113	-2.54	1979
172	LOC433332	433332	217827A	2.4385	-2.54	3469
172	LOC433332	433332	254569A	1.8211	-2.54	5582
173	Rrm2	20135	64497A	3.5748	-2.54	1422
173	Rrm2	20135	150659A	3.5748	-2.54	1453
174	AI894139	101197	176538A	4.2493	-2.54	876
174	AI894139	101197	176538B	2.2258	-2.54	4045
174	AI894139	101197	100279A	1.8211	-2.54	5616
175	6720430O15	241556	168947B	13.179	-2.53	50
176	Scamp4	56214	185337B	7.6217	-2.53	200
176	Scamp4	56214	185339A	2.0235	-2.53	4829
176	Scamp4	56214	185337A	1.8211	-2.53	5641
177	2810406C15Rik	68298	90926B	2.0848	-2.53	4249
177	2810406C15Rik	68298	181271A	2.0235	-2.53	4833
177	2810406C15Rik	68298	181270A	1.8211	-2.53	5649
178	Osmr	18414	68477A	3.695	-2.53	1266

178	Osmr	18414	68665A	3.5748	-2.53	1467
179	Lim2	233187	98880A	3.58	-2.53	1342
179	Lim2	233187	171542B	1.5513	-2.53	8164
179	Lim2	233187	171542A	1.349	-2.53	9490
180	Dfy	13349	66710A	3.5748	-2.52	1448
180	Dfy	13349	161479A	3.5748	-2.52	1473
181	C230086A09Rik	320277	97873B	12.66	-2.52	57
181	C230086A09Rik	320277	97873A	2.0754	-2.52	4283
181	C230086A09Rik	320277	97779A	1.8211	-2.52	5678
182	Zfp451	98403	87205B	13.018	-2.52	52
183	D330014H01Rik	216851	98820A	3.5748	-2.51	1372
183	D330014H01Rik	216851	98820B	2.0754	-2.51	4526
183	D330014H01Rik	216851	98630A	1.8211	-2.51	5726
184	Usp8	84092	74299A	12.971	-2.51	53
185	1110036H21Rik	66182	78155B	4.0469	-2.51	1041
185	1110036H21Rik	66182	182759B	3.5748	-2.51	1503
186	4933434I20Rik	67555	80121A	2.231	-2.50	3718
186	4933434I20Rik	67555	80217B	1.9197	-2.50	5397
186	4933434I20Rik	67555	80217A	1.8211	-2.50	5772
187	4932415D10Rik	237411	167335B	4.0469	-2.50	1047
187	4932415D10Rik	237411	94929B	3.5748	-2.50	1514
188	4921511H03Rik	70920	166087A	8.566	-2.50	133
188	4921511H03Rik	70920	166086B	3.5748	-2.50	1515
189	AI449441	208084	94055B	5.5983	-2.50	457
189	AI449441	208084	93959B	3.5748	-2.50	1523
190	Slc22a13	102570	87577B	12.815	-2.49	55
191	Pde7b	29863	184117A	2.9003	-2.49	2259
191	Pde7b	29863	71827B	2.0235	-2.49	5117
191	Pde7b	29863	72017A	1.8211	-2.49	5822
192	Pax1	18503	63479B	2.2258	-2.49	4156
192	Pax1	18503	63391A	1.9197	-2.49	5308
192	Pax1	18503	63479A	1.8211	-2.49	5832
193	Mtx1	17827	71051A	10.792	-2.48	76
193	Mtx1	17827	156845B	3.5748	-2.48	1549
194	1700049K14Rik	73382	283769B	3.5748	-2.48	1560
194	1700049K14Rik	73382	283770B	2.8017	-2.48	2443
195	Adam4	11498	162345B	3.7771	-2.48	1245
195	Adam4	11498	162346B	2.8017	-2.48	2445
196	Olfr1135	258654	165261A	3.5748	-2.48	1413
196	Olfr1135	258654	288013A	2.7773	-2.48	2446
197	Sn	20612	69425B	2.2258	-2.47	4165
197	Sn	20612	69425A	1.8211	-2.47	5848
197	Sn	20612	151782A	1.8211	-2.47	5897
198	37I24Rik	69612	181169A	3.9951	-2.47	1079
198	37I24Rik	69612	181169B	3.5748	-2.47	1563
199	C130032J12Rik	218975	99884B	3.9951	-2.47	1092
199	C130032J12Rik	218975	171621B	3.5748	-2.47	1571
200	Kif20a	19348	64135B	12.613	-2.47	58
201	Abcb9	56325	74594A	10.59	-2.47	87
202	Nck2	17974	156451B	2.4956	-2.46	3409
202	Nck2	17974	156451A	1.5513	-2.46	7735
202	Nck2	17974	68411B	1.349	-2.46	9977
203	Zfp13	22654	70015A	12.608	-2.46	59
204	Catnal1	54366	184872B	7.2637	-2.46	226
204	Catnal1	54366	73313B	3.5748	-2.46	1591

205	Cd36	12491	160082B	12.452	-2.45	60
206	Tpk1	29807	72004B	12.343	-2.45	61
207	Kcnn3	140493	172447B	12.296	-2.44	62
208	1190002A17Rik	68870	287230A	5.7753	-2.44	419
208	1190002A17Rik	68870	287231A	2.5942	-2.44	3040
208	1190002A17Rik	68870	287231B	1.349	-2.44	10184
209	Klhl8	246293	154637B	4.2493	-2.44	935
209	Klhl8	246293	154638B	3.5748	-2.44	1631
210	1110034A24Rik	109065	172068A	2.698	-2.44	2805
210	1110034A24Rik	109065	172067A	2.2644	-2.44	3651
210	1110034A24Rik	109065	172068B	1.8211	-2.44	6076
211	1700021K02Rik	65971	76661A	3.3725	-2.44	1707
211	1700021K02Rik	65971	76661B	2.2687	-2.44	3629
211	1700021K02Rik	65971	262921B	1.8211	-2.44	6083
212	12M14Rik	76389	77346B	11.933	-2.43	63
213	Set	56086	261251A	2.231	-2.43	3693
213	Set	56086	262923A	1.8211	-2.43	5700
213	Set	56086	262923B	1.349	-2.43	10264
214	Ly6e	17069	62729B	4.9238	-2.42	633
214	Ly6e	17069	155211B	3.4688	-2.42	1657
215	Olfr908	258872	91868B	2.4956	-2.42	3341
215	Olfr908	258872	91938B	1.8678	-2.42	5495
215	Olfr908	258872	91780B	1.8211	-2.42	6148
216	MGC5739	434402	220437A	3.7322	-2.42	1260
216	MGC5739	434402	220437B	3.4688	-2.42	1661
217	9130423L19Rik	74570	87254A	2.2258	-2.42	3860
217	9130423L19Rik	74570	178594A	2.2258	-2.42	3890
217	9130423L19Rik	74570	87345B	1.8211	-2.42	6159
218	Syngr3	20974	151945B	11.871	-2.42	65
219	Nck1	17973	68410A	2.1028	-2.41	4224
219	Nck1	17973	156449A	1.5513	-2.41	7734
219	Nck1	17973	156449B	1.349	-2.41	10381
220	Ndor1	78797	99671B	11.669	-2.41	66
221	F10	14058	61528A	2.0754	-2.41	4377
221	F10	14058	157430A	1.8211	-2.41	5892
221	F10	14058	157429B	1.8211	-2.41	6205
222	Lrrn1	16979	62889B	9.8476	-2.41	100
223	1810061M12Rik	67016	219771A	4.2493	-2.41	888
223	1810061M12Rik	67016	219770B	3.4243	-2.41	1690
224	Tnni3k	435766	97638A	3.9951	-2.40	1086
224	Tnni3k	435766	97543B	3.4243	-2.40	1697
225	Ddefl1	230837	218330A	11.424	-2.40	68
226	Myh14	71960	177319A	2.698	-2.40	2866
226	Myh14	71960	82464A	2.0754	-2.40	4380
226	Myh14	71960	177319B	1.8211	-2.40	6269
227	AB041803	232685	169365A	3.7771	-2.39	1196
227	AB041803	232685	88488A	3.3725	-2.39	1724
228	Psma1	26440	188129B	11.051	-2.38	71
229	Olfr518	258303	262998A	2.2258	-2.38	3908
229	Olfr518	258303	91037A	2.0754	-2.38	4331
229	Olfr518	258303	91130A	1.7641	-2.38	6372
230	Olfr918	258372	91422A	3.3725	-2.36	1716
230	Olfr918	258372	263014B	3.3725	-2.36	1792
231	Olfr577	259113	103220A	10.792	-2.36	75
232	Rassf1	56289	74198B	2.8244	-2.35	2442

232	Rassf1	56289	74198A	2.4089	-2.35	3511
232	Rassf1	56289	185489A	1.7122	-2.35	6512
233	Defb11	246081	88230B	2.4956	-2.35	3315
233	Defb11	246081	103101B	2.0235	-2.35	5022
233	Defb11	246081	88326A	1.7122	-2.35	6520
234	Myoz3	170947	86785A	10.014	-2.35	94
234	Myoz3	170947	86785B	3.3725	-2.35	1809
235	Gpr40	233081	288322B	10.896	-2.35	74
235	Gpr40	233081	261672B	3.3725	-2.35	1812
236	P2ry1	18441	155750A	10.792	-2.35	77
237	Whrn	73750	218936B	2.7498	-2.35	2674
237	Whrn	73750	218938B	2.0235	-2.35	5180
237	Whrn	73750	218936A	1.7122	-2.35	6540
238	BC051227	234384	154802A	9.0064	-2.34	116
239	Acad9	229211	94541B	2.9003	-2.34	2373
239	Acad9	229211	163926B	2.0754	-2.34	4608
239	Acad9	229211	94541A	1.7122	-2.34	6551
240	B930007L02Rik	321006	284223B	10.792	-2.34	78
241	Cd209e	170780	86713B	3.7771	-2.34	1250
241	Cd209e	170780	167062B	2.698	-2.34	2882
242	Flrt3	71436	99519A	2.9003	-2.34	2239
242	Flrt3	71436	99704B	2.698	-2.34	2884
243	4933431E20Rik	329735	168584B	3.5748	-2.34	1520
243	4933431E20Rik	329735	99401A	3.2703	-2.34	1834
244	Rgn	19733	64269B	10.792	-2.33	79
245	A630065K24Rik	213417	88807B	2.9003	-2.33	2294
245	A630065K24Rik	213417	169386B	2.0235	-2.33	5068
245	A630065K24Rik	213417	169388B	1.7122	-2.33	6620
246	Ormdl2	66844	77480B	10.792	-2.33	80
247	Olfr32	18331	261083B	2.0235	-2.33	5099
247	Olfr32	18331	287443A	1.7537	-2.33	6447
247	Olfr32	18331	287444B	1.7122	-2.33	6637
248	2310015I08Rik	56538	74845B	2.8017	-2.33	2444
248	2310015I08Rik	56538	185834B	2.698	-2.33	2924
249	Acsl6	216739	163634A	8.672	-2.33	130
249	Acsl6	216739	88724B	3.2687	-2.33	1862
250	Pcdhb17	93888	175501A	2.2258	-2.32	4007
250	Pcdhb17	93888	85890A	2.0235	-2.32	4867
250	Pcdhb17	93888	85806B	1.7122	-2.32	6670
251	Syt14	329324	168847B	5.5983	-2.32	469
251	Syt14	329324	168846A	1.7122	-2.32	6559
251	Syt14	329324	168845B	1.7122	-2.32	6678
252	Psx2	104384	77462B	10.792	-2.32	82
253	Tbx4	21387	187210A	6.5893	-2.32	296
253	Tbx4	21387	187210B	3.2687	-2.32	1876
254	1700012B18Rik	71839	177258A	3.5748	-2.31	1392
254	1700012B18Rik	71839	177260B	3.2279	-2.31	1886
255	Sfrs14	234373	100857B	10.792	-2.31	83
256	Dlat	235339	152729A	2.2258	-2.31	3997
256	Dlat	235339	152728B	2.0235	-2.31	5133
256	Dlat	235339	152729B	1.7122	-2.31	6716
257	4930548H24Rik	67656	179929B	4.7214	-2.31	718
257	4930548H24Rik	67656	179928B	3.1965	-2.31	1889
258	Enpp1	18605	155845A	4.2493	-2.31	897
258	Enpp1	18605	63602B	2.0235	-2.31	5121

258	Enpp1	18605	155845B	1.7122	-2.31	6718
259	Myadm	50918	72938A	4.0469	-2.31	1002
259	Myadm	50918	73123B	3.1797	-2.31	1906
260	D6Ertd32e	52055	98580A	10.74	-2.30	85
261	3830417A13Rik	70696	258746B	2.9003	-2.30	2324
261	3830417A13Rik	70696	258746A	1.9197	-2.30	5269
261	3830417A13Rik	70696	258745B	1.6716	-2.30	6779
262	Tcl1b1	27379	71750A	2.698	-2.30	2819
262	Tcl1b1	27379	71661A	2.4956	-2.30	3178
262	Tcl1b1	27379	71567B	1.6716	-2.30	6788
263	Tas2r109	387343	257633B	3.4243	-2.30	1689
263	Tas2r109	387343	257634A	3.1701	-2.30	1919
264	C730027P07Rik	245050	99020A	3.1797	-2.30	1893
264	C730027P07Rik	245050	99020B	2.0754	-2.30	4461
264	C730027P07Rik	245050	98924B	1.6716	-2.30	6792
265	30019M04	331537	99451A	10.59	-2.30	86
266	0610006O14Rik	76252	178586B	5.7813	-2.30	418
266	0610006O14Rik	76252	178586A	2.5942	-2.30	3026
267	Aof1	218214	93656A	4.1507	-2.29	958
267	Aof1	218214	170704B	3.1701	-2.29	1934
268	80H04Rik	214058	94214A	10.532	-2.29	88
269	4930529M08Rik	78774	96474A	3.2687	-2.29	1839
269	4930529M08Rik	78774	96563B	1.6716	-2.29	6783
269	4930529M08Rik	78774	176300A	1.6188	-2.29	6855
270	Scd3	30049	186079A	4.9238	-2.29	613
270	Scd3	30049	186078A	3.113	-2.29	1947
271	BC060631	234776	99079A	4.0891	-2.28	990
271	BC060631	234776	99079B	2.5942	-2.28	3070
272	Smox	228608	89727B	10.377	-2.28	89
273	Agtpbp1	67269	76841A	3.5748	-2.28	1434
273	Agtpbp1	67269	179241A	3.113	-2.28	1966
274	2410014A08Rik	109154	173816B	2.8819	-2.28	2409
274	2410014A08Rik	109154	173816A	1.8211	-2.28	5521
274	2410014A08Rik	109154	96647B	1.6188	-2.28	6917
275	BC034834	226143	223140A	2.5942	-2.27	3024
275	BC034834	226143	223140B	2.5534	-2.27	3110
276	F730014I05Rik	228866	90705A	6.0704	-2.27	358
276	F730014I05Rik	228866	90705B	3.113	-2.27	1978
277	AI413414	382056	220223B	10.117	-2.27	92
278	Olfr1511	258268	91182B	2.7773	-2.27	2449
278	Olfr1511	258268	91182A	2.4996	-2.27	3134
279	Adamts13	279028	223120A	3.6319	-2.27	1311
279	Adamts13	279028	223120B	3.113	-2.27	1996
280	Phldb2	208177	165510B	10.117	-2.26	93
281	Pcdhb13	93884	85886A	3.5748	-2.26	1459
281	Pcdhb13	93884	175491B	3.113	-2.26	2002
282	Robo1	19876	152172B	3.6319	-2.26	1334
282	Robo1	19876	152171A	3.1085	-2.26	2013
283	Exosc6	72544	101502B	4.3064	-2.26	859
283	Exosc6	72544	101647A	3.1085	-2.26	2015
284	5930416I19Rik	72440	166464B	3.113	-2.26	1973
284	5930416I19Rik	72440	166464A	3.1067	-2.26	2021
285	A430041E03	235327	98984B	2.9003	-2.26	2290
285	A430041E04	235327	168316A	2.0235	-2.26	4751
285	A430041E03	235327	98984A	1.5565	-2.26	7036

286	B230312A22Rik	230088	94645B	2.9003	-2.26	2298
286	B230312A22Rik	230088	94458B	2.5942	-2.26	3088
286	B230312A22Rik	230088	94645A	1.5565	-2.26	7038
287	Trim26	22670	84805A	10.014	-2.25	95
288	Ankrd16	320816	167987B	10.32	-2.25	90
288	Ankrd16	320816	98282B	2.5942	-2.25	3090
288	Ankrd16	320816	167987A	1.5565	-2.25	7043
289	9230112E08Rik	320800	98279B	4.2493	-2.25	922
289	9230112E08Rik	320800	167975B	3.1027	-2.25	2027
290	Nupl1	71844	93308B	6.3595	-2.25	321
290	Nupl1	71844	178738A	3.0727	-2.25	2038
291	Myo7b	17922	85241A	3.3725	-2.25	1749
291	Myo7b	17922	157160B	2.9003	-2.25	2336
291	Myo7b	17922	85336A	1.5565	-2.25	7084
292	Pdlim7	67399	163001B	4.2493	-2.25	921
292	Pdlim7	67399	79947B	2.2258	-2.25	4047
292	Pdlim7	67399	79947A	1.5565	-2.25	7085
293	Fv1	14349	66855A	9.8476	-2.24	98
294	6030457N17Rik	68079	261271A	8.4311	-2.24	147
295	3230401I01Rik	67417	80047A	2.7498	-2.24	2501
295	3230401I01Rik	67417	179643B	2.2258	-2.24	4057
295	3230401I01Rik	67417	179641A	1.5565	-2.24	7118
296	4930563M21Rik	75258	257493A	7.2171	-2.24	227
296	4930563M21Rik	75258	257493B	3.0205	-2.24	2058
297	4930579K19Rik	75881	102688B	4.4516	-2.24	810
297	4930579K19Rik	75881	102598B	3.0205	-2.24	2059
298	Dffb	13368	160532B	4.9238	-2.24	638
298	Dffb	13368	61240B	3.0205	-2.24	2063
299	Fads1	76267	166247A	9.8476	-2.24	99
300	Cts3	117066	81270A	8.3047	-2.24	149
301	Sec22l3	215474	99785B	5.7072	-2.23	432
301	Sec22l3	215474	99967A	1.7122	-2.23	6491
301	Sec22l3	215474	99785A	1.5565	-2.23	7162
302	Acvr1	11477	162108A	2.2933	-2.23	3586
302	Acvr1	11477	60027A	2.0235	-2.23	4846
302	Acvr1	11477	59932A	1.5565	-2.23	7171
303	H3f3a	15078	61913A	9.738	-2.22	102
304	BC020025	217198	90625B	9.7023	-2.22	103
305	4432409M07Rik	70785	93037B	4.0176	-2.22	1063
305	4432409M07Rik	70785	93224A	2.9055	-2.22	2115
306	Bfar	67118	183766A	1.8211	-2.21	5683
306	Bfar	67118	183768A	1.5565	-2.21	7105
306	Bfar	67118	79359B	1.5565	-2.21	7275
307	D12Wsu95e	217864	171787B	3.4311	-2.21	1665
307	D12Wsu95e	217864	171786A	1.7641	-2.21	6345
307	D12Wsu95e	217864	171788B	1.5565	-2.21	7278
