# Supplementary material for: A high-throughput screen of inactive X chromosome reactivation identifies the enhancement of DNA demethylation by 5-aza-2′-dC upon inhibition of ribonucleotide reductase
Source: Epigenetics Chromatin. 2015 Oct 13;8:42. doi: 10.1186/s13072-015-0034-4 (PMC4604769; doi:10.1186/s13072-015-0034-4)
Supplement: Supplementary file 8 — 10.1186/s13072-015-0034-4 Analysis of autosomal DNA methylation in MEFs treated with combinations of RNR inhibition and 5-aza-2’-dC. A. (i) Heat map of the unsupervised hierarchical clustering as in Fig. 3B but only for autosomal CpG sites within CpG islands (CGIs). Genomic locations of CpG islands were obtained from UCSC Genome browser (see "Methods" section). Constitutively hypermethylated (>0.75) and hypomethylated (<0.15) sites were filtered out to improve contrast. (ii) Heat map of the unsupervised hierarchical clustering as in Fig. 3B but only for autosomal CpG sites within promoters. As in (i), constitutively hypermethylated (>0.75) and hypomethylated (<0.15) sites were filtered out to improve contrast. Promoters were defined as the region 1 kb upstream of the TSS for all UCSC genes. B. Heat maps of the unsupervised hierarchical clustering as in Fig. 3C for autosomal CpGs within CGIs with at least 5X coverage by RRBS across samples, but filtered for sites with methylation levels in the untreated sample of either (i) 0.75–1.0 (ii) 0.50–.75 (iii) 0.25–0.50 or (iv) 0–0.25. C. As in Fig. 3D but for replicate samples. D. Pairwise significance test results conducted using a two-sample Kolmogorov-Smirnov test (‘KS-test stat’ and ‘KS-test p-value’ columns) between the distributions of autosomal CpG methylation in Fig. 3D and Figure S7C, as well as two measures of effect size: Cohen’s d and the differences between these ‘upper modes’ between the comparisons (‘Delta upper mode’ column). [file 13072_2015_34_MOESM8_ESM.pdf]

Minkovsky_SuppFig7

A

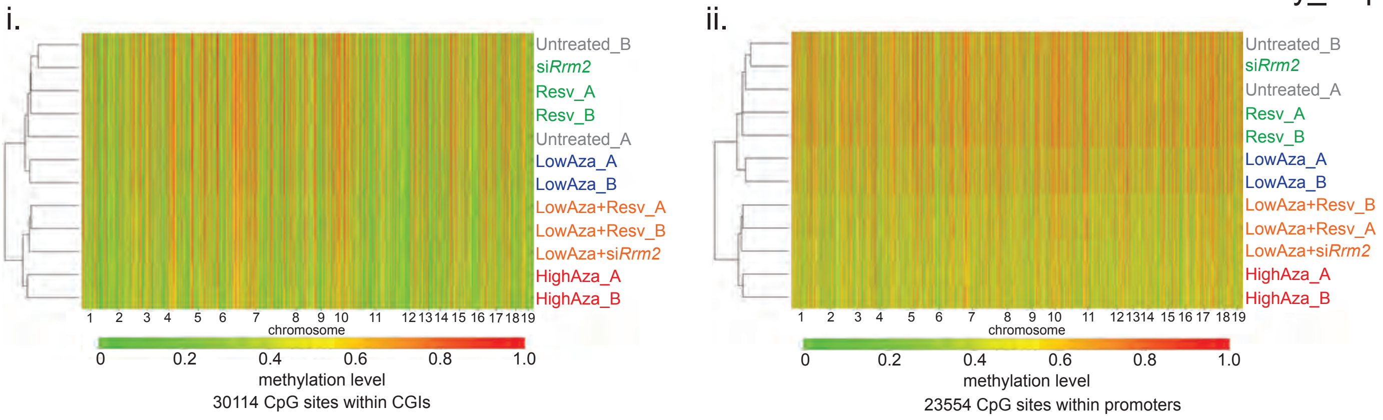

B

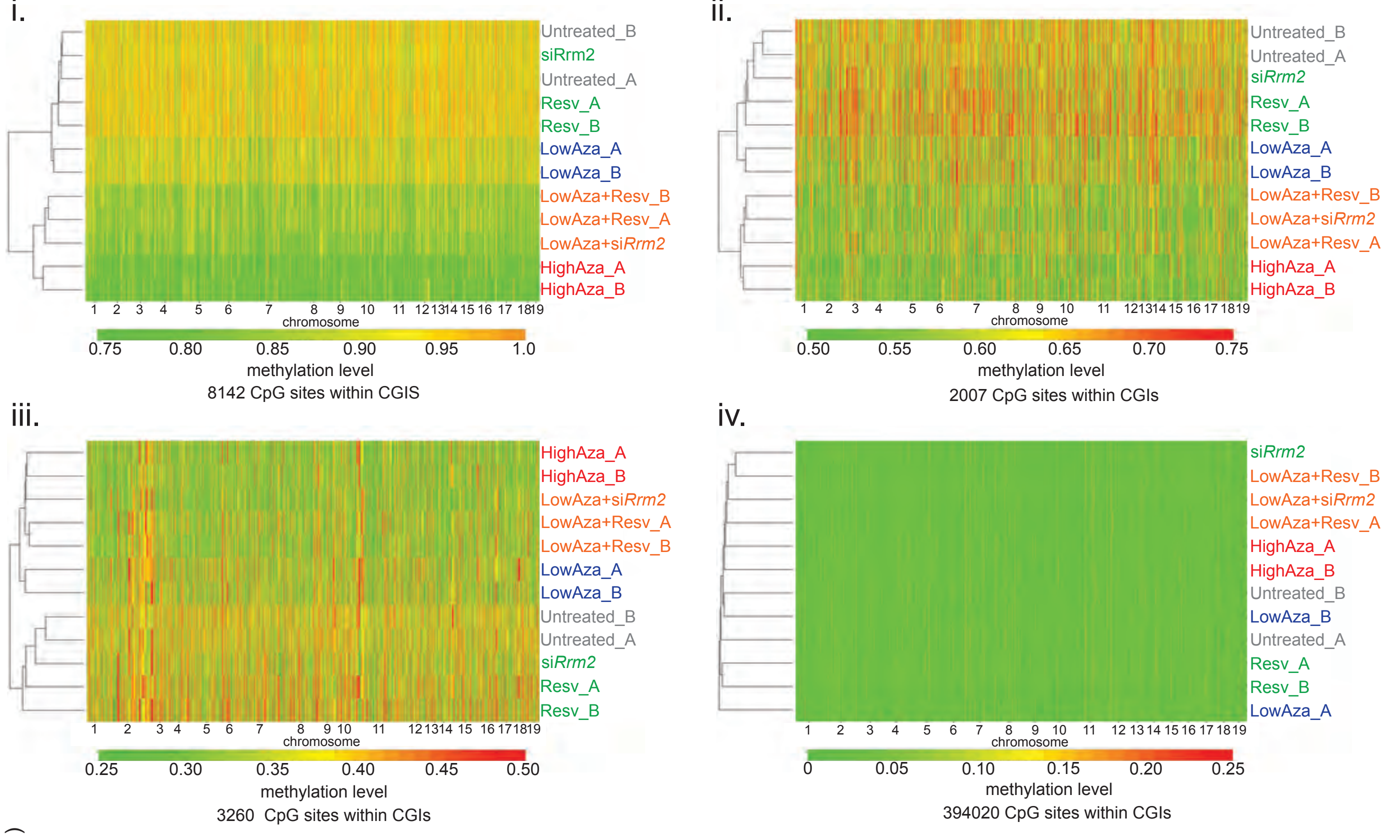

C

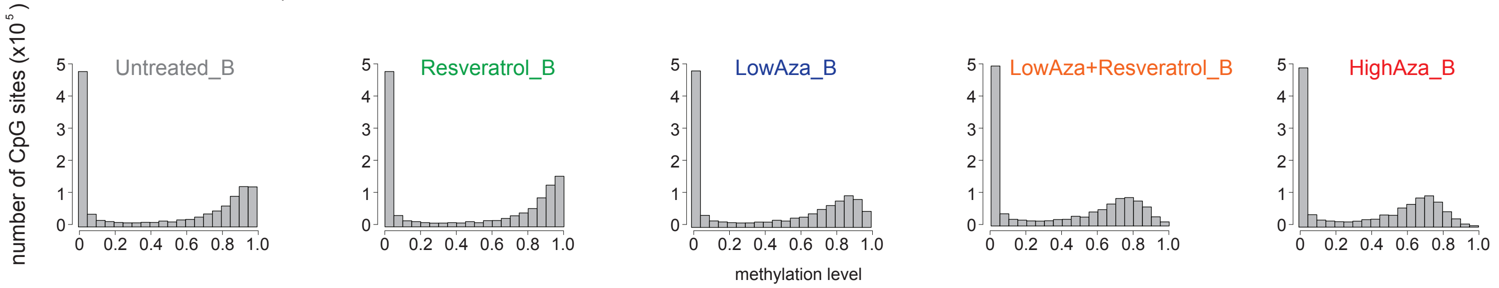

D

Comparison	KS-test statistic	KS-test p-value	Cohen's d	Delta of upper mode
Untreated_A-vs-HighAza_A	0.26	<2.22e-16	0.24	0.25
Untreated_A-vs-HighAza_B	0.26	<2.22e-16	0.25	0.25
Untreated_A-vs-LowAzaResveratrol_A	0.20	<2.22e-16	0.18	0.20
Untreated_A-vs-LowAzaResveratrol_B	0.20	<2.22e-16	0.18	0.20
Untreated_A-vs-LowAzaSiRrm2_A	0.23	<2.22e-16	0.23	0.25
Untreated_A-vs-SiRrm2_A	0.02	<2.22e-16	0.01	0.05
Untreated_A-vs-Resveratrol_A	0.03	<2.22e-16	0.02	0.00
Untreated_A-vs-Resveratrol_B	0.03	<2.22e-16	0.02	0.00
Untreated_A-vs-LowAza_A	0.09	<2.22e-16	0.07	0.10
Untreated_A-vs-LowAza_B	0.09	<2.22e-16	0.08	0.10
Untreated_B-vs-HighAza_A	0.26	<2.22e-16	0.24	0.20
Untreated_B-vs-HighAza_B	0.26	<2.22e-16	0.24	0.20
Untreated_B-vs-LowAzaResveratrol_A	0.20	<2.22e-16	0.18	0.15
Untreated_B-vs-LowAzaResveratrol_B	0.20	<2.22e-16	0.18	0.15
Untreated_B-vs-LowAzaSiRrm2_A	0.23	<2.22e-16	0.22	0.20
Untreated_B-vs-SiRrm2_A	0.02	<2.22e-16	0.01	0.00
Untreated_B-vs-Resveratrol_A	0.03	<2.22e-16	0.02	-0.05
Untreated_B-vs-Resveratrol_B	0.03	<2.22e-16	0.02	-0.05
Untreated_B-vs-LowAza_A	0.09	<2.22e-16	0.07	0.05
Untreated_B-vs-LowAza_B	0.09	<2.22e-16	0.08	0.05
Untreated_A-vs-Untreated_B	0.00	2.79088E-05	0.00	0.05
HighAza_A-vs-HighAza_B	0.01	<2.22e-16	0.01	0.00
LowAzaResveratrol_A-vs-LowAzaResveratrol_B	0.00	2.59E-13	0.00	0.00
Resveratrol_A-vs-Resveratrol_B	0.00	2.55015E-08	0.00	0.00
LowAza_A-vs-LowAza_B	0.00	1.07E-09	0.00	0.00
